# Advances in brain and religion studies: a review and synthesis of recent representative studies

**DOI:** 10.3389/fnhum.2024.1495565

**Published:** 2024-11-29

**Authors:** Patrick McNamara, Jordan Grafman

**Affiliations:** ^1^Department of Psychology, National University, San Diego, CA, United States; ^2^Boston University School of Medicine, Boston, MA, United States; ^3^Center for Mind and Culture, Boston, MA, United States; ^4^Cognitive Neuroscience Lab, Shirley Ryan AbilityLab, Chicago, IL, United States; ^5^Department of Psychology, Feinberg School of Medicine, Northwestern University, Chicago, IL, United States

**Keywords:** neuroscience, religion, spirituality, supernatural agents, REM sleep, dreams

## Abstract

We review and synthesize recent religion and brain studies and find that at a broad network neuroscience level, religious/spiritual experiences (RSEs) appear to depend crucially upon interactions between the default mode network (DMN), the frontoparietal network (FPN), and the salience network (SN). We see this general result as broadly consistent with Menon’s et al. “Triple Network or Tripartite Model” (TPM) of neuropsychiatric function/dysfunction. A TPM cycling model is here offered to account for details of neural bases of an array of RSE phenomena including ecstatic seizures, neuroimaging of religious participants, psychedelically induced mystical states and perceptions of supernatural agents. To adequately account for SA perceptions, however, recent evidence suggests that REM sleep and dreaming mechanisms likely play a role. Future research should examine neurodevelopmental mechanisms of acquired SA perceptions as well as societal-level effects such as brain mediated religious beliefs of in-group cohesion and out-group hostility.

## Introduction

The purpose of this paper is to review and synthesize recent work on the brain basis of religious and spiritual experiences (RSEs). We also propose an integrative framework that can guide future research. The field has in our estimation reached a threshold, such that syntheses of results into testable theories would now be fruitful, and where wider dissemination of these findings would further strengthen the work. We will also suggest new directions for research that can build upon important insights attained in the last 5 years. This essay is not meant to serve as a comprehensive review nor an exhaustive cataloging of all recent work. Rather, we aim to provide the reader with a sampling of representative new research findings on RSE phenomena neglected by previous reviews (such as ecstatic religious seizures, psychedelics and REM sleep processes) that point the way toward new understanding of brain mediation of RSEs. In the first half of the paper we review recent studies within the framework of the Triple Network or Tripartite Network Model (TPM) of brain function, the introduce the TPM cycling model of brain mediation of RSEs and then turn to specific RSE phenomena neglected in previous reviews including ecstatic seizures, REM sleep dreaming and supernatural agent cognitions. We conclude the essay by placing brain and religion studies within the broader biocultural evolutionary framework while suggesting lines of investigation for future work or directions in religion and brain studies.

To focus our review and facilitate synthesis of this area of research, we adopt a minimal and narrow definition of RSEs. RSEs can provisionally be understood as those experiences, beliefs or practices that occur to an individual or group when that individual or group engages in rituals, beliefs or practices directed at, or involving sacralized supernatural agents (SAs). This definition is not meant to capture the full array of experiences deemed RSEs, but it is at least consistent with what we believe most religious studies scholars would point to as recurring and essential elements of many if not most RSEs across time and cultures (Norenzayan, 2013; Pyysiainen, 2009).

But why study the brain basis of RSEs in the first place? Identifying the brain basis of RSEs can illuminate their nature, functional components, and their potential biocultural functions. RSEs need to be studied because they have been fundamental for generation of beliefs and behaviors for most people across most historical epochs (Bellah, 2011). Even today billions of people worldwide [5.8B according to a Pew Survey—Key Findings From the Global Religious Futures Project (Pew Research Center, 2022)] claim to be religious. RSEs, furthermore, can promote pro-social and altruistic behavior and enable group-level cooperation (Kelly et al., 2024). But as history also tragically illustrates, religious beliefs and behaviors can also fuel conflict on a truly terrifying scale. RSEs therefore need to be studied and better understood.

The central role of the brain in religious phenomena follows from its central role in human life history. Briefly put, *the* key life history trait among human beings, i.e., the trait that influences all other life history variables in the standard catalog of life history traits (e.g., average lifespan, body size, length of juvenile period, gestational period, interbirth intervals, fertility rates, parenting and aging) is brain size, structure, and function. Gestational period, dependency period, interbirth intervals and parenting effort are all substantially influenced by the need to invest huge metabolic resources in brain development, maintenance, and daily functioning. Among humans, for example, it has been estimated that about 65% of all resting energetic expenditure is used to support the maintenance and growth of the brain in the first year of life (Holliday, 1986). In the adult, the brain requires about 20–25% of the energy of the whole body just to maintain its resting state activity levels despite representing only 2% of the body mass (Chen and Zhang, 2021). Task demands routinely increase the brain’s energy demands up to 10% (Shulman et al., 2014). The central role of the brain in human life history trajectories is especially dependent upon development and functioning of these resting state networks. These functional brain networks even in the resting state exhibit distinct energetic signatures, consistent with their central status in brain function as well as their separate functional specializations (Shokri-Kojori et al., 2019). For example, Shokri-Kojori et al. measured two dimensions of energy requirements of the brain. The first labeled “relative power (rPWR)” captured the level of concurrent metabolic need and observed activity, relative to the rest of the brain. The second dimension labeled relative cost (rCST) captured the extent to which glucose metabolic needs exceed (or fall behind) the observed activity, relative to the rest of the brain. The authors found that that default mode network (DMN) had the highest rPWR while the frontal–parietal network or FPN had the highest rCST or relative cost in energy requirements. We will see below that these 2 brain networks (along with the salience network or SN) play a disproportionately significant role in RSE phenomena.

## Previous reviews of brain and religion studies

**Figure 1:**
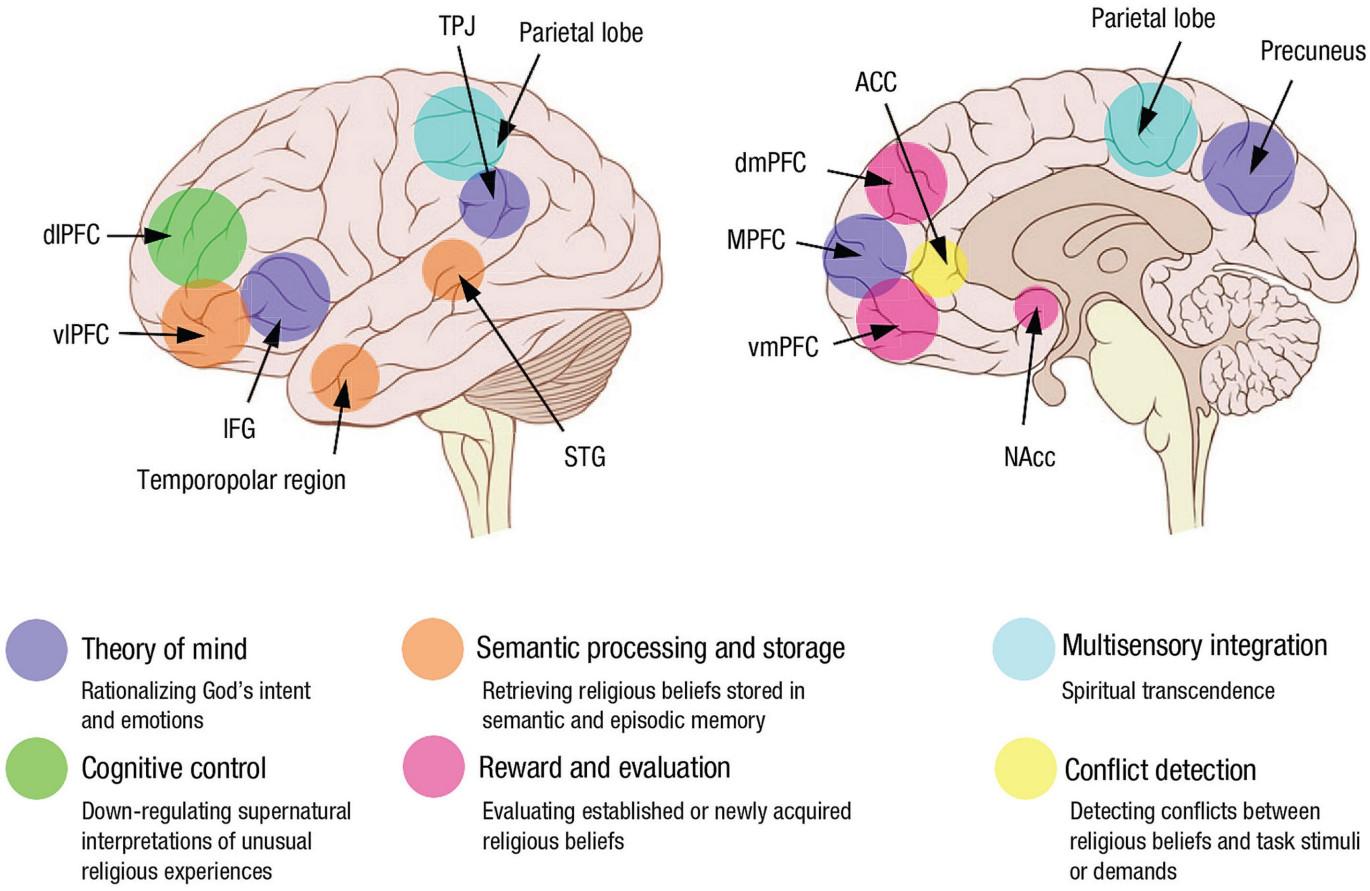
Key brain regions involved in religious beliefs and the social, cognitive, and emotional processes mediated by these structures (used with permission from Grafman et al., 2020). Convergent evidence from functional neuroimaging, noninvasive brain stimulation, and lesion-mapping studies identified a set of brain networks important to religious cognition. The theory-of-mind network consists of the inferior frontal gyrus (IFG), medial prefrontal cortex (MPFC), temporoparietal junction (TPJ), and precuneus and is involved in rationalizing God’s intent and emotions. The semantic-processing and storage network consists of the ventrolateral prefrontal cortex (vlPFC), superior temporal gyrus (STG), and temporopolar region and is involved in retrieving religious beliefs. The cognitive-control network encompasses the dorsolateral prefrontal cortex (dlPFC) and is involved in down-regulating supernatural interpretations of unusual religious experiences. The reward and evaluation network consists of the nucleus accumbens (NAcc), ventromedial prefrontal cortex (vmPFC), and dorsomedial prefrontal cortex (dmPFC) and is involved in evaluating established or newly acquired religious beliefs. Multisensory integration, processed in the parietal lobe, is crucial for experiencing spiritual transcendence. Finally, the conflict-detection and error-response network consists of the anterior cingulate cortex (ACC) and is involved in detecting conflicts between religious beliefs and task stimuli or demands.

In their review of the neuroscience of RSE literature up to about 2016, van Elk and Aleman (2017) noted that existing findings up to then were largely correlational. They tentatively assigned mediatory functions of RSEs to large-scale brain networks known to support non-RSE-related basic cognitive functions. For example, the default mode network (DMN) likely mediated self-transcendent experiences. The components of the so-called “theory of Mind or ToM” network (which is considered a subnetwork of the DMN and includes the face-processing area, the vmPFC or ventromedial prefrontal cortex and elements of the limbic system) mediated prayer and over-attribution of intentionality. The temporal cortex typically associated with language and memory processing mediated mystical and ecstatic experiences, and the anterior cingulate cortex (ACC) and the PFC were associated with top-down mechanisms involved in acquiring, reflecting upon and maintaining intuitive supernatural beliefs. Grafman et al.’s (2020) review in 2020 largely concurred with van Elk and Aleman’s findings but described the relevant networks in information processing terms. Grafman et al. noted that the DMN-related theory-of-mind network is involved in rationalizing God’s intent and emotions. The semantic-processing and storage network consisting of the ventrolateral prefrontal cortex (vlPFC), superior temporal gyrus (STG), and temporopolar region is involved in retrieving religious beliefs. The FPN is involved in down-regulating supernatural interpretations of unusual religious experiences. The salience network (SN; described below) is involved is involved in evaluating established or newly acquired religious beliefs. Multisensory integration, processed in the parietal lobe and the temporal–parietal-occipital junction (TPO), is crucial for experiencing spiritual transcendence. Finally, the conflict-detection and error-response network consists of the anterior cingulate cortex (ACC) and is involved in detecting conflicts between religious beliefs and task stimuli or demands (Figure 1).

Our review of the recent literature largely agrees with these 2 past reviews but in addition suggests that at a broad network neuroscience level, RSE phenomena appear to depend crucially upon interactions between 3 centrally important resting state neural networks: the default mode network (DMN), the frontoparietal network (FPN), and the salience network (SN). We see this general result as broadly consistent with Menon, 2011 and Menon et al., 2023 “Triple Network or Tripartite Model” (TPM) of neuropsychiatric function/dysfunction (see also McNamara, 2022). Bringing in TPM theory into the religion and brain field significantly deepens our understanding of religion and brain relationships by situating these relationships firmly within the wider context of network neuroscience. Viewing RSE phenomena from within the lens of TPM also helps to illuminate specific causal mechanisms, functional capacities and aberrations in religious and spiritual phenomena. However, the devil is in the details and we will see that TPM-level explanations of RSE phenomena, while an advance over previous frameworks, will need to be supplemented by other data and networks to account for existing neuroscience findings concerning RSE phenomena.

## The triple network model

**Figure 2:**
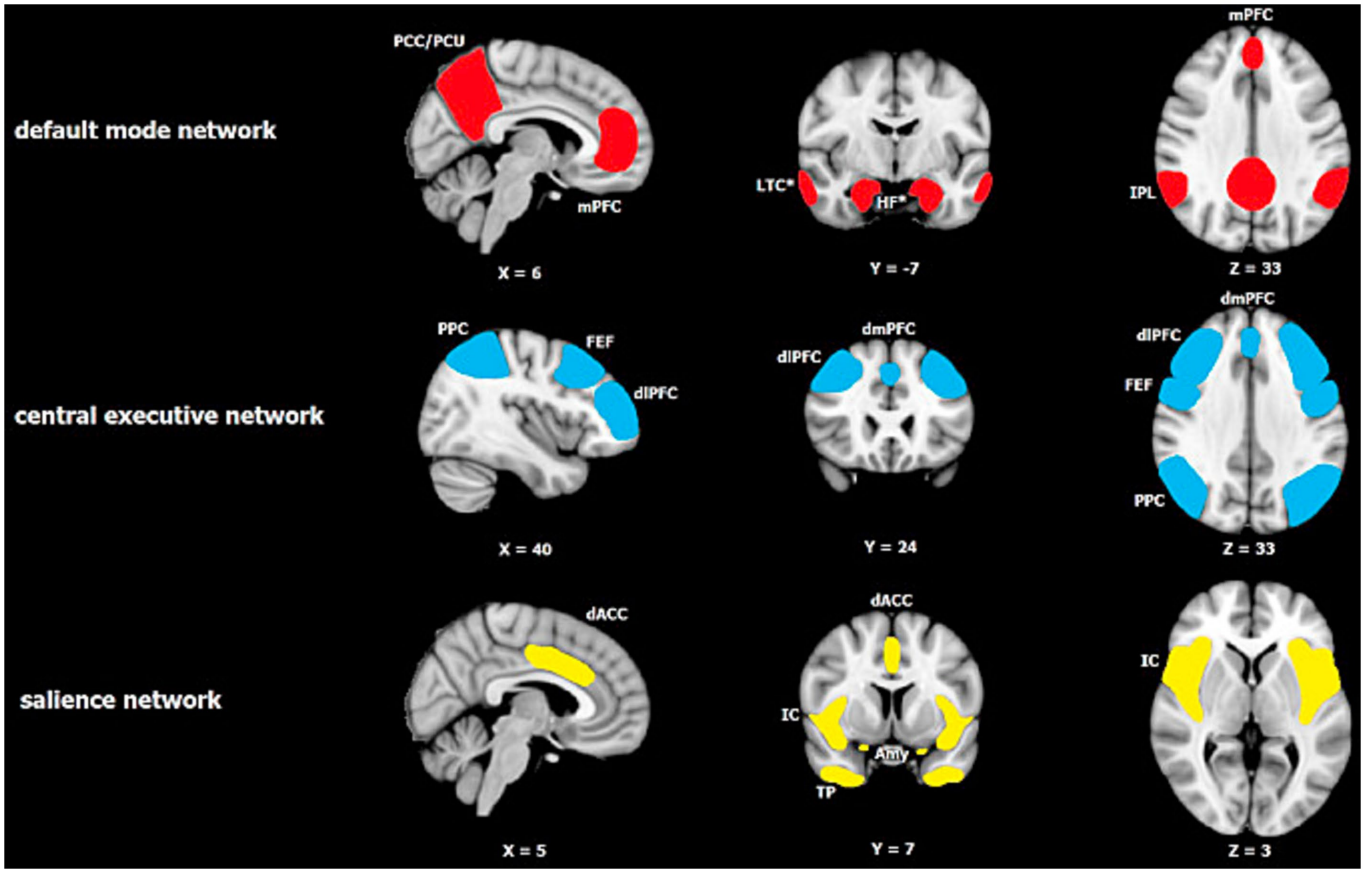
Major resting-state networks relevant to Menon’s Tripartite Model of Brain Function in neuropsychiatric disorders (reprinted with permission from Mulders et al., 2015). The default mode network (DMN) consists of two core regions: the medial prefrontal cortex (mPFC) and the posterior cingulate cortex/precuneus (PCC/PCu), with the inferior parietal lobule (IPL) also being reported consistently. The central executive network (CEN) or what we call the FPN in the paper is centered on the dorsolateral prefrontal cortex (dlPFC) and posterior parietal cortex (PPC), and also includes the dorsomedial prefrontal cortex (dmPFC) and frontal eye fields (FEF). The salience network consists of the insular cortex (IC) including the anterior insular cortex (AIC), dorsal anterior cingulate cortex (dACC), temporal pole (TP) and amygdala (Amy).

The basic idea behind the TPM is that altered activation patterns between 3 canonical resting state neural networks, FPN, DMN, and SN (see Figure 2) are consistently implicated in a range of neuropsychiatric disorders, and thus may be centrally important for neurocognition more generally. In this paper we suggest that TPM, with appropriate adjustments, can also account for a range of RSE-brain associations as well. At this point we need to say a bit more about each of these 3 networks that, we will see, repeatedly appear in the story of religion and brain studies.

## The DMN

The default mode network (DMN) is a highly correlated network of brain regions comprising medial prefrontal cortex (mPFC), lateral superior and inferior frontal gyrus (IFG), rostral anterior cingulate, posterior cingulate cortex (PCC), precuneus (PCu), retrosplenial cortex (RSC), posterior inferior parietal cortex (IPC), angular gyrus, temporoparietaloccipital junction (TPO), temporal pole, hippocampus, parahippocampus, and lateral temporal cortex. The DMN is often conceived to be composed of 2 subsystems: one centered on the hippocampal formation (medial temporal lobe) referred to as the “theory of mind” or simulation subsystem and the other centered on the vmPFC referred to as the self-referential subsystem (Pace-Schott and Picchioni, 2017). DMN activity reflects the brain at rest and is primarily dominant during daydreaming, “mentalizing or theory of Mind tasks” (ToM), future-oriented thinking and most especially self-related experiences and self -referential thought.

FPN activity is significantly negatively correlated with the DMN (Uddin and Menon, 2009), and involves interactions between the lateral posterior parietal cortex (PPC) and the dorsolateral prefrontal cortex (dlPFC; Seeley et al., 2007). The FPN handles what are typically referred to as “executive cognitive functions” (ECFs) such as goal-directed thought, planning, problem-solving, focused attention and reflective thought.

The SN includes the anterior insular cortex or AIC, the dorsal cingulate cortex (dACC; Sridharan et al., 2008) and perhaps the posterior cingulate cortex (PCC) though some authors would place PCC with the DMN. The SN often recruits subcortical nodes in the hypothalamus, thalamus, midbrain and some authors include brainstem nuclei. It is also connected to dopaminergic reward systems including the NAC-meso-limbic and the striatal-prefrontal systems. As mentioned above the SN (according to the TPM) evaluates significance levels of incoming external and internal stimuli and serves as a dynamic “switch” between the DMN and FPN, in line with importance of the information and cognitive effort required to address the challenge.

Interestingly, the SN, particularly its AIC node, is distinguished by a unique cellular component, the von Economo neurons (VENs) (Banovac et al., 2021). VENs are characterized by large spindle-shaped cell bodies with a single basal dendrite. They are high conduction velocity, long range projection and specialized neurons that are often called the “empathy cells” because their degeneration is associated with diminished empathy for others, self-awareness, social cognition, moral reasoning, and emotional intelligence (Sfera et al., 2024). Some cases of bvFTD (especially when the right-temporal lobe exhibits marked degeneration) that are especially characterized by involvement of the SN, are associated with a transient hyperreligiosity (Chan et al., 2009; Veronelli et al., 2017; Block and Miller, 2020).

While the SN is especially characterized by VENs, the SN, and the other 2 networks in the TPM (DMN and FPN) exhibit disproportionate distribution of 5HT2A receptor signaling systems. Relevant to RSE phenomena these signaling systems, in turn, are preferentially modulated by lysergic acid (LSD), psilocybin, and mescaline, which cause the subjective experiences of a psychedelic trip as well as outright hallucinations (Vollenweider and Preller, 2020). 5ht2A antagonists and inverse agonists, including second-generation antipsychotics can block a psychedelic experience if the individual is administered a serotonergic psychedelic and they can reverse the perceptual disturbances of psychiatric illnesses such as schizophrenia (Halman et al., 2024). These 5ht2A receptor signaling systems are also known to regulate sleep mechanisms (Landolt and Wehrle, 2009; Zhao et al., 2022). This fact will become important later when additional brain systems, in this case the REM dreaming system, are brought in to help explain people’s perceptions of supernatural agents (SAs).

Within the TPM framework the SN is theorized to operate as a kind of controlling switch that, depending on task demands, either simultaneously activates DMN and deactivates FPN or vice versa. The FPN and SN demonstrate correlated activity, and both networks are anti-correlated to the DMN. When the salience network increases its activation level after identifying a significant or unexpected/surprising event, it reduces the activity of the self-oriented and mind wandering default mode network and activates the external goal-oriented FPN to deal with the surprise. For tasks demanding focused attention activating FPN and downregulating DMN is required, whereas for tasks requiring self-referential thought or imaginative simulations more generally, relative downregulation of SN and FPN, and upregulation of DMN will be required and so on. Therefore, key aspects of cognitive processing will depend upon proper assignment of relevance or salience to stimuli coming from within the individual or from without the individual. Failing to assign appropriate levels of significance to something important or assigning any level of relevance to something unimportant can create equally dysfunctional cascade effects where the FPN and DMN do not engage or disengage appropriately.

## TPM and RSEs

Given that assignment of salience or importance to experiential stimuli is fundamental for derivation of “meaning” “value” and “significance” to those experiences, it is clear that such a mechanism (as modeled by the TPM) should be of central relevance to religion and spirituality. After all most people conceive religion and spirituality as being centrally concerned with ultimate or sacred values, and individual and group related meaning and purpose in life (Park, 2005). It follows therefore that at a minimum, people will deem an experience or belief spiritual or religious to the extent that it is considered salient, meaningful and significant. In short, to the extent an event is surprising the chances that the event will be deemed an RSE will increase. These assertions or predictions are consistent with recent predictive processing accounts of religion put forth by several authors (Schjoedt, 2009; Van Elk and Aleman, 2017; Andersen, 2019) but predictive processing is not enough to account of RSE phenomena. The predictive processing account of neurocognitive function suggests that the brain operates as a prediction machine which generates models of what is to be expected in the world. When those expectations are violated or when the system is surprised, an error signal is generated which prompts the system to update its predictive models of the world. Given that survival and flourishing depends on the ability of those predictive models to accurately anticipate opportunities and threats it follows that information/events are considered significant and valuable to the extent that they trigger intense surprise and force radical updating of predictive models. These are the kinds of experiences which we claim increase the odds of those experiences being deemed RSEs.

If we understand the TPM framework to be suggesting that when surprise occurs (and the SN is activated) the experience has to be evaluated both reflectively (via FPN) and with respect to significance for the self (via DMN), then it follows that the TPM system must be involved in updating of predictive models. We suggest that such updating involves repeating cycles through the evaluation process such that the salient/surprising error signal is evaluated in relation to self (emphasizing DMN activation) *in the absence of reflective thought* and then switches to evaluating the event more abstractly and reflectively (via FPN) *without reference to the self per se* and so on repeatedly until the event is adequately processed and predictive models are adequately updated. We call this conjecture of the predictive processing enriched version of TPM the “TPM cycling” account of RSEs. We next review the recent representative studies in religion and brain studies in light of this TPM cycling account of RSEs.

## TPM cycling applied to religion and brain studies

The TPM cycling framework arguably illuminates results coming from neuroimaging, clinical neuroscience and experimental neuroscience accounts of changes in RSEs. One recent neuroimaging study suggests that people who regularly attend religious services have significantly different brain connectivity patterns than people who do not regularly attend religious services (Kieckhaefer et al., 2023). Capitalizing on the neuroimaging and behavioral data from ∼40,000 participants from the UK Biobank population cohort, Kiekhafer et al. used structural and functional fMRI brain scan data gathered on these participants to explore how social participation, including weekly attendance at religious groups, is reflected in the human brain. When contrasted with people who frequented social clubs or sports groups, people participating in religious groups were especially characterized by increased volumes in pre and postcentral gyrus, right middle temporal gyrus, right superior temporal gyrus and right rostral anterior cingulate –all sites within SN or DMN. Overall, the authors reported a combination of enhanced within-network and between network functional connections within the DMN, SN, and in the FPN cognitive control network. By contrast, the dorsal attention network and the visual cortex both showed reduced within network connectivity strengths in what they called “spiritually active” people relative to the other social groups. Clearly, if regular religious participation influences resting state brain functional networks this report places that influence firmly within the TPM with “spiritually active” people exhibiting enhanced connections both within each network and between all 3 networks relative to people who regularly attend other non-religious social activities such as sports clubs. Religious people apparently tend to utilize or cycle through the TPM more regularly than others.

TPM also appears able to account for clinical RSE phenomena. For example, for some patients with bipolar disorder (BPD) religious states and perceptions of supernatural agents (SAs) increase during a manic episode and become absent during a depressive episode. Although the neurobiology underlying bipolar disorder is exceedingly complex there is some evidence linking changes in the SN and in dopaminergic transmission to changes in religious beliefs in the two major phases of the illness—the manic vs. the depressive phases. Dopamine is a catecholaminergic neurotransmitter involved in a wide array of physiological functions but is known to be crucial for voluntary movements and reward salience-among other cognitive functions. Excessive dopaminergic stimulation, for example, via administration of dopamine agonist such as amphetamine, can trigger manic symptoms in patients with BPD (Beaulieu and Gainetdinov, 2011; Brunello and Tascedda, 2003; Anand et al., 2011). Conversely, depression is associated with a hypo-dopaminergic state (Ashok et al., 2017). In a recent review of the literature Ouwehand (2024) found that many patients with BPD report intense spiritual experiences during the manic phase but largely lose these experiences during the depressive phase. For example, during the manic phase the BPD patients tended to report sensing God’s presence as if he was actually sitting in same room as the patient, or they reported that they felt a heightened sense of meaning and purpose etc. During the depressive phase the patients reported a feeling the absence of God, and an increase in religious doubt, guilt and suicidal ideation.

Recent meta-analytic studies of neuroimaging studies of bipolar disorder patients have shown that functional integration within and among the three TPM core brain networks (SN, DMN, and FPN) is abnormal (Sha et al., 2019; Zhang et al., 2023). BPD patients evidenced altered connectivity both within networks (FPN, SN) and between (DMN-SN, DMN FPN). In particular, during the euthymic and manic stages hyperconnectivity within the FPN and reduced connectivity between SN and FPN and SN and DMN were noted (Zhang et al., 2023). In the depressive stage connectivity within FPN and between FPN and other networks was reduced. Thus with the case of BPD we get clear RSE experiential changes as a function of both phase of the illness and differences between connectivity patterns within and between the 3 canonical networks of the TPM. If there is reduced connectivity between SN and DMN/FPN during the manic phase then presumably SN cannot effectively act as a switch that down regulates DMN and up-regulates FPN thus leaving the DMN overactivated resulting in a grandiose sense of self without critical reflection from FPN input. As significance increases in association with the sense of Self so does the tendency to ascribe religious significance to that experience. There is reduced input from FPN so checks on that ascription process are inhibited. In the depressive phase the TPM system itself is not functioning so all meaning-generation is lost and the individual loses contact with his or her experience of God.

TPM can illuminate findings from experimental brain stimulation studies of RSEs as well. For example, in a pre-registered study, Holbrook et al. (2020) reported that while death or threatening primes transiently increases religious beliefs, downregulating posterior medial prefrontal cortex or pMFC via cTBS (transcranial brain stimulation) *reduces* self-reported religious belief after those beliefs had been stimulated by a recent, prime concerning death and bodily decomposition suggesting that you need pMFC –a key node within the DMN, for those religion-promoting primes that are related to threats to the sense of self to work. Similarly, Nencha et al. (2022) found that generation of an ecstatic religious state in a 56 year old male with intractable epilepsy depended upon electrical stimulation in the dorsal AIC –a key node in the SN. Electrical stimulation in the dorsal AIC repeatedly produced a blissful sensation of mental clarity, without any epileptiform after discharge on the SEEG. The patient’s self-reported scores on the mystical experiences questionnaire (MEQ) significantly increased after AIC stimulation and he reported that “everything seems simplified.” He felt “liberated” and reported a very strong feeling of fullness. His consciousness “had suddenly enlarged”; “it is like looking at infinity, I no longer have any limits, as if everything was connected, and I was connected with any part around me.” He started to describe these feelings 1 to 2 s after the end of the 3-s stimulations. The authors suggested that the blissful state was due to electrical stimulation preventing the generation of interoceptive prediction errors via inhibition of AIC activity. In addition to acting as the central node in SN operating as a regulator of FPN and DMN interactions, the AIC may also act as a comparator of expected- to -actually experienced interoceptive and emotional reactions. When that comparator suggests that all expectations have been met and that there are no surprises to deal with, then spiritual bliss and mystical states ensue.

We see a similar phenomenon occurring in so-called ecstatic seizures, described as mystical or spiritual states by the patients themselves. Dostoyevsky may have experienced these sorts of seizures (Hughes and Melyn, 2005). In their review of the neurology of ecstatic auras/seizures whose related experiential states were dubbed mystical or religious by the patients themselves, Gschwind and Picard (2016) studied 52 published cases. They concluded that the ecstatic symptoms in these patients appeared to localize to a functional network centered around the AIC. Geschwind and Picard note that patients with these kinds of seizures often report that “everything seems to be exactly in the right place, as it should be,” that perfect solutions have been arrived at, that all is well and all will be right with the world, and that this is a spiritual insight and RSE. The authors hypothesize that “in the ecstatic auras the mechanisms of interoceptive prediction error generation are blocked, the comparator between the actual and the predicted state no longer functions, and therefore there is no more mismatch” (Gschwind and Picard, 2016, p. 14) and thus the patient concludes that all is right with the world and all is right between him and God etc. If we evaluate Geschwind and Picard’s account of ecstatic seizures against the TPM framework we might conclude that the switching/appraisal mechanism, the SN, is appraising the information it is receiving as highly significant possibly because the AIC within the SN is returning no error / surprise signals. Therefore, the SN would presumably not need to engage the DMN and FPN. There is no challenge/surprise to meet and therefore there is no need to engage/disengage these networks. Over time, however, there presumably would be downstream effects on FPN and DMN along with corresponding effects on RSEs. But only longitudinal studies with such patients would yield answers to the questions posed by these patients.

With respect to religion-related seizure activity, the TPM cycling account of RSE phenomena may throw new light on clinical observations that arguably launched the field of religion and brain studies back in the 1970s (Dewhurst and Beard, 1970; Waxman and Geschwind, 1975; Geschwind, 1979; Bear and Fedio, 1977). These reports described a small group of patients with a particular form of temporal lobe epilepsy (TLE) who seemed to ascribe extraordinary meaning and significance to their interictal experiences. These patients deemed many of their experiences as RSEs and often wrote reams of material to expound on the religious significance of their insights. In attempting to explain such phenomena Bear and Fedio (1977) argued that the tendency to ascribe intense significance to everyday experiences was due to hyper-connectivity (due to seizure activity) between cortical sites mediating language comprehension with subcortical sites such as the limbic system and the amygdala which mediate emotional processing. Some patients with TLE had greater connectivity between these cortical and subcortical sites and therefore inappropriately assigned greater significance to incoming sensory events. The fact that the patients were ascribing too much significance to their everyday experiences can be accounted for by either a chronically activated SN network or by SN over-activating DMN and down regulating FPN thus emphasizing the overwhelming significance of events for the sense of Self. And the hypergraphia or filling notebooks with reams of metaphysical speculations on these experiences suggests an over-activated FPN-related reflective processing on those experiences. In short, when something like the Geschwind syndrome of hyper-religiosity, hypo-sexuality and hypergraphia occurs in a small number of patients with TLE, it may be mediated in part by this TPM cycling phenomenon when individual experiences are being repeatedly and dysfunctionally examined in relation to self and then over-reflected upon.

The TPM cycling account is also consistent with (1) the relation of belief formation to meaning making processes in religion; (2) the core group of findings concerning religion’s facilitation of emotional and physical healing; (3) the relation between religion and empathy and (4) most recent “4E” accounts of brain/mind relations.

### TPM and belief formation

With regard to the relation of belief formation to meaning making processes in religion we rely on our own work (Krueger and Grafman, 2012; Cristofori et al., 2022) and the work of Seitz et al. (2018). According to the Seitz et al.’s, credition model, beliefs are the result of neural processes involving perception, valuation, information storage, and prediction. They are considered unique probabilistic cognitive representations based on sensory signals from external and internal sources of noise and then built up via social interactions involving rituals and construction of social narratives. Formally, Seitz et al. suggest that this basic model can be stated as: B=S/N * V.

A belief (B) is probabilistically and dynamically formed over time by the perception (P) of an object or event, that is, by identification of a signal (S) out of a sample of ambient noise (N). The larger the S/N ration the greater confidence expressed in the belief. But every belief is also theorized to be assigned a level of salience, significance or value, V, for the individual. The value can be either positive (indicating reward or attraction) or negative (indicating aversion, threat, or danger). In addition, S/N and V are independent of each other; thus, a large S/N can have a small V, and a small S/N can have a large subjective V. Thus, in general, a positive value of B is an indicator of a meaningful representation of high probability, that is, a “belief. Seitz et al. note that the entire belief formation process tends to be regulated by medial PFC with the valuation process in particular being dependent upon mPFC. In our previous review of brain networks crucial for religious belief formation Cristofori et al. named the prefrontal cortex including dlPFC and vmPFC, and more posterior regions such as superior temporal cortex (STC), and pSTS/TPJ (default mode network).

Both the Seitz et al. credition model and the Crisfori et al. model of the neural processes involved in belief formation are consistent with the TPM cycling account presented here given that the SN is explicitly assigned the task in the TPM (as in the credition and Cristofori models of valuation or assessment of the significance of experiences). The TPM cycling account adds to these models of belief formation the additional process of the elaboration of meaning via ritual and social contexts via engagement of the FPN in particular and the concomitant downregulation of the DMN.

### TPM and religion’s effects on health

There have been several exhaustive reviews of the literature (Koenig et al., 2024; Dornisch, 2024) on religion’s effects on measures of health. While thousands of studies have now been published many of these studies are methodologically flawed, but an increasing number of rigorous studies, in this area, have been published. Both the older literature and the recent more methodologically sound studies suggest reliable protective effects of religious participation, especially religious service attendance, on health and mental health, for outcomes as diverse as all-cause mortality, depression, suicide, cancer survival, and subjective well-being. With respect to the neural basis of these effects Rosmarin et al. (2022) evidence indicates that S/R is associated with protective mental health-related neurobiological correlates, including cortical thickness, decreased default mode network (DMN) connectivity, and greater posterior alpha. We note in passing that decreased DMN connectivity and increased posterior alpha power are also brain signatures associated with both REM sleep and the serotoninergic psychedelics, both of which we discuss below. In their review of the literature on neural correlates of R/S, van Elk and Aleman (2017) suggested that temporal brain areas are associated with religious visions and ecstatic experiences; multisensory brain areas and the default mode network are involved in self-transcendent experiences; the Theory of Mind-network (including mPFC) is associated with prayer experiences and over attribution of intentionality; top-down mechanisms instantiated in the anterior cingulate cortex and the medial prefrontal cortex could be involved in acquiring and maintaining intuitive supernatural beliefs.

The literature on religion and health is partially consistent with the TPM cycling account offered here. But to see that clearly we have to bring in the evidence from psychedelics and REM sleep neurobiology and the role these play in the salience and default mode networks. We discuss these matters more thoroughly below in the sections on REM sleep and psychedelics. Briefly put, optimal mental health depends on the balance between the correlated activity levels of the 3 major networks within the TPM. If the DMN is chronically over-activated it will inhibit FPN functioning and thus gradually impair mental functioning. Conversely if the DMN is chronically under-activated similar mental health decrements will occur. Mental health problems in turn gradually make it more difficult to cope with chronic health issues.

### TPM cycling and empathy

Empathy, or the ability to feel and sympathize with the pain and suffering of another has always been linked with morality and has often been actively promoted by religions. Empathy very likely is crucial for facilitating prosocial behaviors and therefore larger scale cooperative enterprises. In their review of the literature on the neural basis of empathy He et al. (2024) noted crucial roles for the anterior and middle cingulate cortex (ACC, MCC), middle and anterior insula cortex (MIC, AIC) as well as prefrontal and sensorimotor regions. Within this network, the cingulate and insula primarily extract the affective dimension of pain and interact with the subcortical, motor, and prefrontal regions to appraise meaning. In neuropsychiatric disorders associated with impaired empathy He et al. found that the left anterior cingulate gyrus, adjacent medial prefrontal cortex, and right middle temporal gyrus, were dysfunctional. The hyperactive regions showed network-level interactions with the core default mode network (DMN).

As mentioned above from the TPM cycling perspective optimal mental health, including normal empathic responding, depends on the balance between the correlated activity levels of the 3 major networks within the TPM. If the DMN is chronically over-activated as appears to be the case where dysfunction in empathic responding is documented, it will inhibit FPN functioning and thus gradually impair mental functioning. Conversely if the DMN is chronically under-activated similar mental health decrements will occur.

### TPM cycling and 4E

In our previous work (e.g., McNamara, 2022) we noted that religious beliefs and experiences likely behave much like any other ordinary beliefs and experiences. But beliefs and experiences are the product of brain-culture nexus that we call “the mind.” But to understand the Mind we adopt and assume some, not all of the claims of “4E cognitive science” (suitably modified to take into account relevant brain functioning and constraints), namely that the mind is embodied, enacted, embedded and extended. It is embodied insofar as it reflects and expresses bodily needs and aims. Mind does not float out there in some nebulous cultural ether untethered to body and brain. Our basic cognitive capacities and categories are grounded in bodily sensorimotor experiences and elaborated via metaphor. Many computations performed by the brain begin with internal or interoceptive messages or information. These bodily computations scaffold more complex representations such as the sense of self. The mind, furthermore, is enacted. It is built for action, for grasping and manipulating the objects and things in the world. It is not some passive information processing device. It is always operating in a world. Its beliefs and models of the world are oriented toward actionable goals. But this does not, as some enactivists have argued, imply or mean that the brain does not process computations, content or representations. There is abundant evidence that the brain does indeed perform computations on what can be construed as very rich cognitive content/representations. Mind, however, is indeed embedded in the world. Mind manifests only when interacting with the world or particular contexts. No ecological context-no mind. Finally, Mind is therefore extended. It does not end at the skin of the thinker/doer. It extends into the context, the environment. Our tools, for example, are extensions of our minds and mind, in turn, are shaped and extended by the tools. The tools themselves are extensions of the culture. The upshot with respect to religion of these 4E considerations is that the brain does not “contain” the religious mind. The religious mind is not reducible without remainder to the brain. The brain is necessary for manifestation of the religious mind but the mind itself has to be thought of as larger than the physical brain. It may be partially co-extensive with the culture, but it interacts with culture which then operates on the brain eliciting some mental capacities and shutting down others. These capacities then articulate with the cultural processes of other minds and artifacts to produce “my mind.” Without culture there would be no mind, but without brain there would be no culture. Historically, religion utilized these pre-existing cognitive capacities in order to promote cumulative cultural evolution and to facilitate human cooperation and group success via conflict between religiously defined out-groups. All that the TPM cycling account can add to the 4E framework on the religious mind is that optimal meaning construction and social interactions occur when TPM cycling is operating normally as discussed more thoroughly below.

Given the brief summary above of some representative studies in brain and religion, and the relation of important topics in religion to the TPM cycling account offered here, the TPM cycling framework looks promising for characterizing the religious influences on brain network functioning. It nevertheless remains unclear whether the neural basis of core aspects of RSEs—namely encounters with spiritual or “supernatural agents” (SA) such as God or angels or demons etc., can be so captured. We turn next to that core aspect of RSE phenomena: supernatural agent cognitions.

## Brain systems necessary for perceptual encounters with supernatural agents/SAs

Early work identified the DMN/ToM networks as especially important for cognizing SAs. For example, Kapogiannis et al. (2009, 2014) using Granger Causality analytic techniques on brain scan data from religious vs. non-religious participants identified a large number of brain areas within the DMN network that activated when participants verified statements concerning the involvement of God in daily life. Significantly, a pathway from the right inferior frontal gyrus (IFG) was demonstrated to modulate a network downstream from it when participants were evaluating statements about daily interactions with God —namely the dorsomedial prefrontal cortex (dmPFC) and precuneus, − brain regions previously found to be involved in reward salience, value judgments, self/other processing and emotional memory. This causal effect of IFG on downstream networks held true only for religious individuals. The brain sites modulated by these religious statements concerning interactions with God all were situated within the SN or DMN. These data seem to suggest that encounters with SAs are related-to, or extensions of normal ToM mentalizing processes we regularly utilize to model the minds of others. However, this simple account situated as it is within the TPM, may not be the whole story. When we look for neural-causal mechanisms generating SA percepts rather than mere correlations between brain activity patterns and religious cognitions, the story gets more complicated.

**Figure 3:**
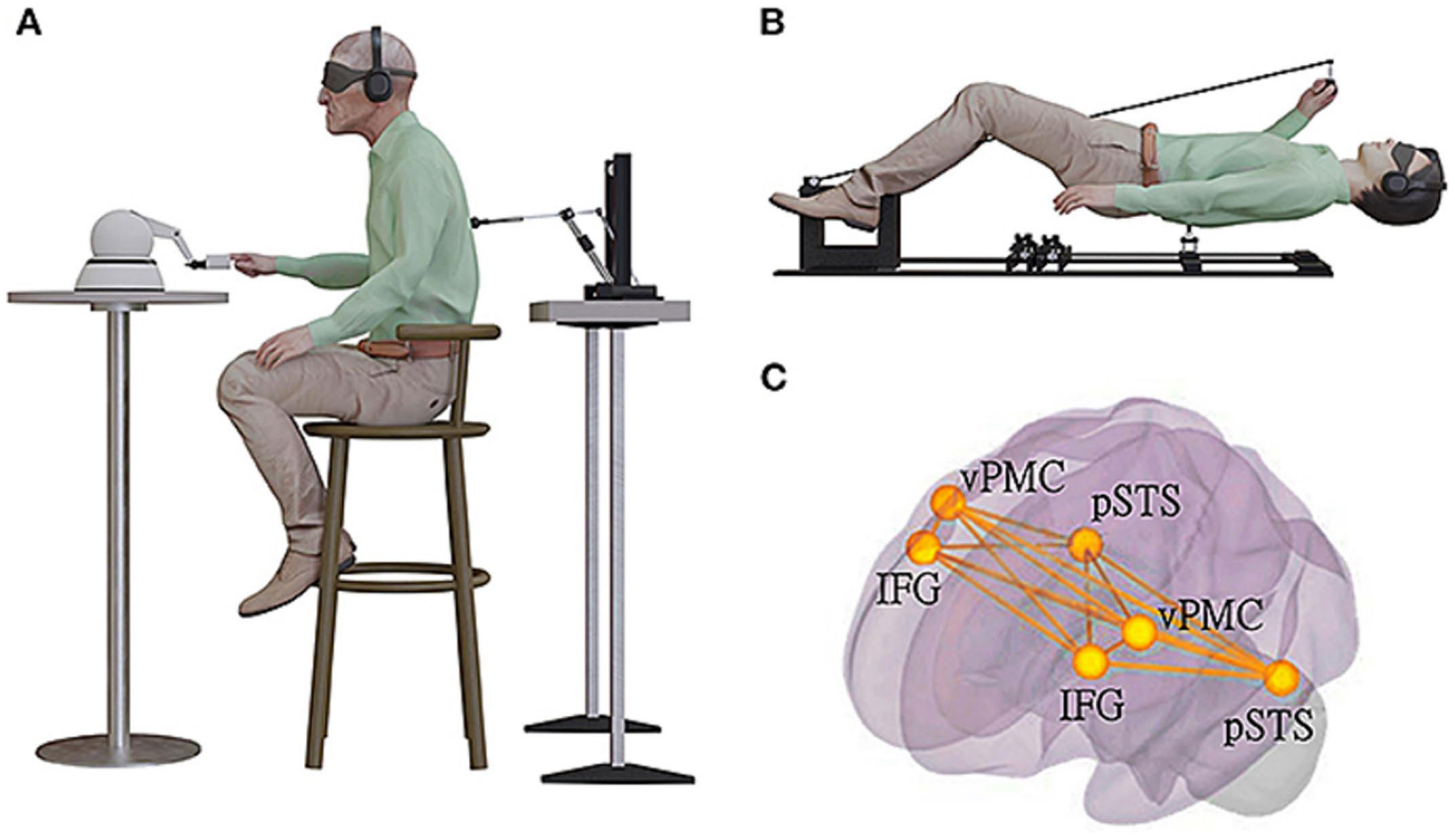
Experimental set up to induce sense of presence of an unseen agent (used with permission from Vehar et al., 2022). (A) The robotic set-up used for induction of invisible presences in patients with Parkinsons’ disease in a sitting position. Patient was moving the front robot in a poking motion, using the right-hand index finger, while receiving a corresponding tactile stimulation on their back. They were in a state of sensory deprivation, wearing headphones playing white noise and with their eyes closed, wearing a blindfold. The stimulation on the back was either synchronous with their movement of the front robot (the back robot had 0 ms of delay) or asynchronous (the back robot randomly delayed from 0 to 500 ms in steps of 100 ms), with the asynchronous stimulation being significantly associated with experiencing robot-induced PH (ri-PH) as a function of sensorimotor delay. (B) The robotic set-up was adapted to be MR-compatible and used for an fMRI study in a healthy population of participants. (C) The brain activation and connectivity patterns were collected in an fMRI experiment with healthy and neurological non-parkinsonian patients. The schematic bilateral display of the connectivity overlap between the network connectivity in spontaneous PH identified using lesion network and connectivity analysis and ri-PH network from healthy participants. The bilateral regions are ventral premotor cortex (vPMC), inferior frontal gyrus (IFG), and posterior superior temporal sulcus (pSTS).

Vehar et al. (2022) described an experimental neuroscience set-up which can be used along with fMRI to reliably induce perception of an unseen presence /SA or what they called “presence hallucinations” or PH. Ronchi et al. (2018) had previously developed a robotic stimulation system that exposed participants to temporally and spatially conflicting sensorimotor signals. Participants were asked to repeatedly move a small robot in front of them with their hand (motor, tactile, and proprioceptive signals) while receiving tactile feedback on their back delivered by another small robot in back of them (back robot), under conditions of sensory deprivation (Figure 3A). Being subjected to such conflicting somato-sensory-motor stimulations characterized by an additional delay between front and back robot elicits PH in healthy individuals. Such an experimental set up fools the cognitive system into inferring the presence of an agent other than the self or patient/participant.

Given that PH or the sense of an invisible agent appears when the self-generated motor signal (to move robot in front of participant) occurs within 500 ms of being stimulated on the back by the robot behind participant, Vehar et al. (2022) suggest that the sense of an unseen presence or PH comes from an error or mis-match signal between an expected sensation and what actually occurred. The participant expects feedback sensations to occur immediately after he moves the front robot. When that expected sensation does not occur and instead the back is stimulated, surprise ensues and an error signal is generated. To account for the error signal the mind generates a hypothesis that an unseen agent must be responsible for the feedback. In short, the authors argue that invisible presences result from faulty integration of motor signals with somatosensory signals. These somatosensory-motor signals are summated at the cortical level within IFG, TPO, AIC and posterior superior temporal sulcus (pSTS) into the global self-representation of a person’s body (Park and Blanke, 2019). Now, once again, all of these sites are situated within canonical TPM networks—particularly the SN and DMN networks. The suggested neurocognitive mechanism producing the SA percept is that when the mismatch between expected sensation and actual outcome occurs there is a misperception of oneself as another agent because the mind has to guess at what caused the mismatch and then generates another agent as the most likely explanation for the error signal. When the error signal occurs the SN presumably considers that a salient signal and then activates the switch to down regulate DMN and upregulate FPN in order to evaluate the error signal. The neuroimaging data from Vehar et al. suggests that engagement of TPM networks mediate generation of perception of the PH or unseen agent/SA. Apparently, when the TPM oscillates between DMN downregulated/ FPN upregulated and FPN downregulated, DMN upregulated, the TPM system with its ToM simulation capacities and the FPN system with its abstracting capacities generates hypotheses/guesses or explanations as to why the surprise occurred and then offers the conclusion that an unseen agent was responsible. Down-regulation of DMN is causal because it results in surprise when an event occurs and the individual cannot conclude that he or she is the agent of the event (given that sense of self is reduced when DMN is reduced). The error signal triggers SN to start the TPM evaluation cycle which then concludes with the claim that if the event cannot be traced back to the individual (and it cannot because sense of self is degraded when DMN activity is inhibited) then an unseen agent is responsible for the event.

## Entity encounters under psychedelics

As with an experimental set-up such as Vehar et al. design, we can also assess causal effects within the nervous system with pharmacologic agents. Stimulation of brain networks via administration of serotonergic psychedelics results in dose–dependent alterations in self-consciousness, experiences of self-transcendence, and crucially for religious cognition: encounters with other-worldly entities including some that could be characterized as supernatural agents or SAs such as “spirit guides,” angels, demons and religious figures. While anecdotally it appears that most people who have taken a psychedelic do report encounters with SAs, we can learn about those encounters via interviewing people who actually claim such experiences. For example, in two investigations of people who encountered SAs while on a psychedelic (*N* = 561, Davis et al., 2020; *N* = 606, Griffiths et al., 2019) 96% asserted that the SAs encountered were conscious beings, intelligent (96%), benevolent (78%), sacred (70%), and agentic (they had special powers) (54%). Majorities of respondents firmly believed that the SA existed independently of their consciousness, i.e., that there were “realer than “real” beings. Approximately 28% of the sample reported that they were atheist before the entity encounter, but there were only 10% of respondents who self-reported as atheist after the encounter.

What might the brain mechanisms be that could produce such profound encounters with SAs? As we have just seen from the Vehar et al. (2022) experiments the TPM networks appear to be involved and mismatch error signals around the issue of individual intentional states, agency or generation of actions are crucial. With respect to generation of SA perceptions during psychedelic experiences similar mechanisms appear to be involved. In an early landmark study, Carhart-Harris et al. (2012) found that psilocybin decreased cerebral blood flow in the thalamus, putamen, hypothalamus, posterior cingulate cortex, precuneus, bilateral angular gyrus, supramarginal gyrus. Anterior cingulate cortex, frontoinsular cortex and orbitofrontal cortex. Most of these structures involve the DMN or the SN. The importance of the DMN and SN to the psychedelic experience was later confirmed by others. For example, Tagliazucchi et al. (2016a, 2016b) utilized fMRI to investigate “ego dissolution” correlations after global and local brain connectivity changes from the resting state following intravenous injection of LSD versus placebo in 15 healthy volunteers. The “ego dissolution” experience is important to investigate as it appears to be crucial for generating the full psychedelic experience. In particular, it predicts the onset of mystical experiences, presumably including SA encounters during the trip. Tagliazucchi et al. (2016a, 2016b) found that the DMN and the thalamus showed *increased* global connectivity under the drug but *decreased* within-network integrity. Lebedev et al. (2015) studied brain functional connectivity correlates of ego dissolution and mystical experiences in 15 volunteers given 2 mg of psilocybin. Analysis of within-network integrity revealed an association between decreased salience network integrity and ego dissolution. In addition, ego dissolution was also associated with decreased functional connectivity between the hippocampus and both the SN and the FPN. The intensity of ego dissolution was found to be strongly anticorrelated to the density of connections in the anterior part of the para-hippocampal circuit or PHC. The PHC, in turn, mediates the connectivity of the hippocampus with the DMN. Thus, the result of down regulation of the PHC is decoupling between structures in the medial temporal lobe (including hippocampus) and the DMN. In short decreased resting-state functional connectivity within the SN and between the PHC and prefrontal cortex was identified under psilocybin and was predictive of ego dissolution (Carhart-Harris and Nutt, 2017). Overall, psychedelics appear to decrease intrinsic DMN connectivity but extrinsic connectivity patterns between DMN and other networks are generally increased and in addition SN activity is altered (Gattuso et al., 2023; van Elk and Yaden, 2022; Stoliker et al., 2022). Stoliker et al. (p. 879) suggest that “Decreased DMN connectivity likely relates to ego dissolution and may involve the dissolution of self-related priors (beliefs) sustained by the DMN. These priors may support a narrative sense of self that disintegrates during ego dissolution”.

What initiates the psychedelic experience to begin with appears to be activation of 5HT2A receptor signaling systems. The classical serotoninergic psychedelics show high affinity for these receptors and if these receptors are pharmacologically blocked there will be no psychedelic experience even if a psychedelic is administered. The 5-HT2A receptor is involved in learning and memory, pain perception, and the sleep/wake cycle (Duerler et al., 2022). 5-HT2A receptors can be found in the so-called pyramidal neurons in layer 5 of the cortex, and in transmodal, higher-order association areas in the brain, such as the temporo-parietal junction and the medial prefrontal cortex—once again sites firmly within the DMN and thus the TPM framework. However, the strongest concentration of 5HT2A receptors may be found in the striate and extrastriate visual cortices (Preller and Vollenweider, 2018)—sites not accounted for within TPM. In any case, the wealth of findings associated with the renaissance in psychedelic studies suggest that activation of 5HT2A receptor signaling systems in SN, DMN and extrastriate and striate cortex may be related to, or permissive for SA cognitions.

But the TPM framework does not readily account for recruitment of extrastriate visual systems under psychedelic conditions of ego dissolution/mystical states and via activation of 5HT2A receptor signaling systems. Both the TPM networks, and the visual systems, however, are known to be recruited during REM sleep which in turn is tightly regulated by 5HT2A signaling systems. Indeed, recent work by various investigators in the religion and brain field have suggested an important role for REM sleep and dreams in generating RSEs—especially encounters with SAs.

## A potential role for REM sleep and dreams in RSE phenomena

One of the most striking cases for the perception of an SA occurs in sleep paralysis. Sleep paralysis (SP) is a transient experience of conscious paralysis when transitioning into sleep or out of sleep (Hishikawa, 1976). REM sleep is associated with atonia or paralysis that lasts the entire REM episode but occasionally the paralysis persists into the waking state so the person is conscious but cannot move. A recent meta-analysis (Hefnawy et al., 2024) involving 76 studies from 25 countries with 167,133 participants estimated the global prevalence of SP was 30%. Basically, a third of all persons likely have experienced at least one and probably several episodes of SP. Subjective phenomena associated with SP involve perceptions of “intruders” or a “succubi” sitting on the chest of the victim. These SAs are often influenced by cultural scripts around the supernatural and considered as demonic SAs who intend the paralyzed victim harm (Cheyne et al., 1999). What brain mechanisms mediate perception of an SA in some cases of SP? Since the majority of the SAs in SP are perceived to be malevolent and threatening one possibility is that SP is mediated by brain circuits within the limbic system dedicated to detection of threats which gets triggered during an episode of sleep paralysis. The paralysis sets up an expectancy of impending threat. That recruits the threat detection system which then triggers some other part of the brain which attempts to explain the source of the threat and concludes it must be an evil presence with super-normal powers. That other part of the brain that generates the explanation for the experience of threat we suggest are the TPM networks DMN and FPN as described under the TPM cycling account. But can TPM cycling account for other SA phenomena associated with sleep and dreams?

REM sleep and dreams more generally appear, in fact, to be very potent sources of SA perceptions. Take the case of so-called “visitation dreams” for example. About 50 to 60% of hospice patients report a “visitation” by someone –a relative or a spirit guide who is not there while they dream or are awake: a phenomenon renowned in end-of life care as a deathbed vision or End-of-Life Dreams and Visions (ELDV; Rabitti et al., 2024). Conversely, many (up to 80% in some estimates) bereaved individuals experience extraordinary visitations of their deceased loved ones in a dream (Wright et al., 2013). The bereaved individual often experiences the deceased individual in the dream as “realer than real,” and absolutely “there” or fully present such that the dreamer can smell, feel, touch and embrace the deceased person as if they were alive again. The deceased often imparts a message to the individual that they are OK and not to grieve. The dreamer awakens feeling absolutely convinced that they received a communication from their deceased loved one in the afterlife and thereby proving to them that there is an afterlife.

It should not be surprising that dreams are a very potent source for SA perceptions. Dreaming is a universal human experience. Most recalled dreams likely come from “rapid eye movement” or REM sleep. Rapid eye movement sleep is a state characterized by occurrence of rapid eye movements (REM), electroencephalographic activation comparable to waking consciousness, muscle atonia, hippocampal theta oscillations, and ponto-geniculo-occipital (PGO) waves, which are field potentials that are similar to “orienting reactions” generated after a challenge/threat/surprise. They are generated from the cholinergic brain stem and pons, lateral geniculate nucleus, and striate/extrastriate cortex, and vivid dreaming. 5HT2A receptors and GABA-ergic (*γ*-aminobutyric acid-ergic) neurons localized in the ventrolateral periaqueductal grey function to modulate REM-on neurons. In addition, tract tracing studies in monkeys have demonstrated that VENs project to the PAG and parabrachial nucleus (PBN), indicating a role for VENs in regulation of PAG-related and sleep related functions (Saleh et al., 2017). Thus, PAG and likely 5HT2A receptors are key neural systems implicated in REM onset, maintenance and intensity (Luppi et al., 2017; Park and Weber, 2020). Interestingly, recent work suggests that PAG plays a key role in expressions of RSEs. Ferguson et al. (2022) used lesion mapping analysis and 2 independently assembled datasets –a neurosurgical dataset (*N* = 88) and (penetrating traumatic brain injury dataset, *N* = 105), to examine relationships between self-reported religiosity and lesion site. The authors found that lesions associated with spirituality and religiosity mapped to a human brain circuit defined by connectivity to the periaqueductal grey (PAG). As noted above, the PAG, in turn, is known to play a critical role in descending pain inhibition (it is rich in opioid receptors), autonomic nervous system function, behavioral responses to threatening stimuli and maintains dense interconnectivity with key TPM structures of the SN, DMN and FPN.

Brain mechanisms associated with REM dreaming are clearly consistent with production of mental simulations of counterfactual, bizarre and extraordinary SAs. During REM dreaming external sensory input is reduced, cholinergic and dopaminergic activity levels are high, while serotoninergic and noradrenergic levels are low or absent, thus enhancing brain plasticity and pushing the brain-mind into a visually and emotionally driven, hyper-associative state (Hutchison and Rathore, 2015; Llewellyn, 2016; Pace-Schott and Hobson, 2002; Walker and Stickgold, 2006). In addition, brain regions dynamically modulated during REM tend to strictly obey principles of TPM: fMRI and rCBF scanning with simultaneous EEG scalp recordings of healthy participants asleep in the scanner demonstrate selective activation of the pontine tegmentum, thalamic nuclei, several limbic elements including the amygdala and the hippocampus, anterior cingulate cortex, mediobasal prefrontal lobes, and extrastriate cortex (Dang-Vu et al., 2010; Maquet et al., 1996; Fox et al., 2013). In contrast, the dorsolateral prefrontal cortex and parietal networks are downregulated in REM sleep (Hobson et al., 2000; Nofzinger et al., 1997; Pace-Schott and Picchioni, 2017) just as they appear to be during the psychedelic experience. In non-REM phases of sleep there is a functional uncoupling of the DMN’s anterior and posterior nodes (particularly the MPFC and PCC) which are then re-coupled with the onset of REM (Chow et al., 2013; Horowitz-Kraus et al., 2017). In short, the SN apparently activates DMN elements and down regulates FPN in REM sleep as required by TPM theory.

In summary, REM appears to promote intense visual simulations of socio-emotional associative processing largely in the absence of reflective thought. Thus, experiences of encounters with extraordinary beings in extraordinary realms are not uncommon in dreams and are generally accepted uncritically in our dreams. But do these dreams influence waking consciousness and waking behaviors? Do they influence waking RSEs?

Balch et al. (2023) constructed an experimental design to answer just such questions. They used computational textual analytics on 35,000 dreams and 20,000 religious experience narratives and found that when people report high “supernatural content” including encounters with SAs and extraordinary realms it was experienced subjectively by the dreamer as a set of images invested with high dominance (the image rivets the attention), arousal and negative affect and thus reduced the dreamer’s subjective sense of self-agency. Overt SAs appeared in supernatural environments characterized by strangeness, negative affect, bizarreness, increased sensory elements and insubstantiality. After thus characterizing cognitive features of supernatural content in dream and RSE reports, the authors then recruited *N* = 116 community volunteers to participate in a two-week intensive longitudinal assessment of mood, religiosity measures, stress, social conflict, dream recall and sleep architecture measures. Sleep architecture was monitored with the DREEM – 3 Headband device. Multi-level modeling analyses were conducted with lagged and cross-lagged effects estimated thus enabling exploration and evaluation of causal effects on generation of SAs in dream content. The authors were able to collect 1,502 dreams during the 2 week in-home study from 116 participants. On average participants reported 9 dreams per person across the 2 week period. Construction of computational word dictionaries resulted in identification of dream narratives with high supernatural content. These were judged via unbiased research assistants and AI tools as significantly more strange, bizarre, unpleasant, and containing lesser amounts of dreamer agency than non-supernatural control narratives. Daily stress activity levels predicted subsequent increases in supernatural content in dreams. In addition, the higher the average REM%, the higher the daily subjectively experienced closeness to God ratings by the participant. Dream supernatural content and dreamer agency predicted closeness to God ratings with a 4 day lag but not vice versa. Negative dream affect predicted increases in authoritarian God image ratings also with a 4 day lag. The authors concluded that high supernatural content occurs both spontaneously and can be triggered by daily stressors. The authors concluded that supernatural content is common in dreams and is associated with otherworldly realms and SAs and increases daytime closeness to God ratings. The REM dreaming system may act facultatively to produce positive or negative God images depending on local context (e.g., high levels of stress and uncertainty, low levels of social trust etc.).

## RSEs, SA encounters and social cooperation

The significance of these findings on REM dreams and the generation of positive and negative God images during REM that actually affect daytime spiritual experiences (e.g., closeness to god rating) is due to the fact that one’s unconscious “God image” operates as a central organizing feature of one’s basic religious experience and life narrative (Mencken et al., 2009). If one’s image of God is of a loving, kind and compassionate figure then one’s religious experiences and story will on average be positive. Conversely, a harsh, judgmental and authoritarian image of God will tend to generate greater spiritual struggles, guilt and anxiety. There is now solid evidence that people’s conception of God dramatically influences one’s willingness to cooperate with other, willingness to cheat on cooperative games, mental and physical health as well as a variety of well-being outcomes (Dezutter et al., 2010; Schaap-Jonker et al., 2002; Nguyen et al., 2015; Gall and Guirguis-Younger, 2013). Silton et al. (2014) found that people who believe in an “angry god” are statistically more likely to show signs of suffering from mental disorders. Froese and Bader (2008) show that an harsh, judgmental, authoritarian image of God is negatively related to political tolerance and positively related to support for gun ownership and defense spending. Mencken et al. (2009) present data that suggests that belief in a loving, forgiving God can build bonds of trust, while beliefs in a judgmental, authoritarian God tend to make believers more wary of others. Francis (2007) presented evidence for a correlation between God images and images of self and images of others. Images of God as the God of mercy may be reflected in a more positive self-concept and in higher levels of empathy with and for others, while images of God as authoritarian may be reflected in less positive self-concept and in lower levels of empathy with and for others.

Given that basic God images can influence willingness to cheat on cooperative games, social interactions and levels of social trust Balch et al. (2023) speculate that the REM dreaming system may act to generate God images that facilitate social cooperation depending on social context. If local social cooperative activities are ongoing and successful and the individual is living in a high-trust society then the REM dreaming system would generate God images that are either authoritarian enough to maintain such high trust levels or would produce loving God images. If however there are low levels of social trust in the dreamer’s local environment then the REM dreaming system would be biased to produce more harsh, threatening, frightening, nightmarish and judgmental authoritarian God images. This is because such God images would act to police and punish bad social-actors such as non-cooperative free-riders (see Shariff and Norenzayan, 2011). The so-called supernatural punishment hypothesis (SPH; Johnson, 2016) on the evolution of God concepts in general suggests that an all-seeing, all-knowing, wrathful and judgmental God is watching you to ensure that you do not break the rules of your social group. But creating an authoritarian God image via REM sleep dreaming processes may not be enough to inhibit free-riding over the long term. Other neuro-cognitive mechanisms will need to be recruited to adjust individual behaviors once “sins” or deviations from group moral norms are flagged via the “policemen in the head” mechanism.

## Future directions

As the above summary suggests cognitive neuroscientists have begun to bring to bear powerful techniques such as brain imaging techniques (fMRI, MEG, high density EEG) and neuromodulation approaches (TMS, BCI, DBS) to identify how the brain shapes religion and vice versa. As this work proceeds the outlook is good that new insights will be uncovered as to how religious culture protects against disease, promotes in-group cooperation and out group hatred and elicits the most extreme states of saintliness and fanaticism known to human beings. However, to realize the promise of this new area of research we need to build upon the insights gained in past work.

One area where religion and brain studies are deficient is understanding brain mechanisms that mediate the “groupishness” of religion. How does the brain support group-level aspects of the religious experience? By pursuing this question we may be able to uncover how the brain supports both social cooperation and social conflict. For example, to build upon our understanding on how the brain supports religious group-individual interactions and religious in-group vs. out-group interactions we suggest more work needs to be done on the role neuroendocrine compounds such as oxytocin/vasopressin play in group formation. Oxytocin is known to modulate social affiliation and feelings of belonging and is beginning to be recognized as a major factor in human sociality, serving as the neuroendocrinological hub for social cognition (Dunbar, 2020; Churchland and Winkielman, 2012; Crespi, 2016; Shamay-Tsoory and Abu-Akel, 2016; Stockly, 2020).

To rigorously investigate brain mechanisms of religiously-defined group interactions we suggest we need to remember that religion is a biocultural phenomenon. We therefore need to bring in frameworks from biological anthropology to help us understand the brain’s role in supporting individual vs. group interactions. We humans are experts at creating various types of groups in order to cooperate with one another for some larger purpose. Religion, and religious ritual in particular, has facilitated cooperation at every stage in recorded human history. Dunbar (2020) has suggested that religion was especially good at supporting group cohesion as group size increased over time. Religion according to Dunbar likely evolved in two stages: an early immersive form with no formal structure based on trance-dancing (a form still evident in the rituals and practices of many hunter-gatherers) and a later form which had more formal structures and gave rise to our modern doctrinal religions. The evolution of both internal group cohesion and cooperation between individuals and groups more broadly can be partially understood via inclusive fitness theory, indirect reciprocity/evolutionary game theory, and multilevel selection theory (Wilson and Wilson, 2007). Inclusive fitness refers to investment in genetically-related others. Indirect reciprocity basically involves self-interested give and take with non-related others. Multilevel selection refers to the fact that selection pressures can under certain circumstances occur at the group level as well at the individual/organismic level. Group effects are most clearly seen when groups are involved in direct conflict and competition with other groups. Culture, including religion, is another evolutionary force affecting all levels of the multi-level selection process as well as the formation of distinctive human groups. The dual inheritance model of human evolution refers to two interacting processes in the evolution of human groups: biological evolution and cultural evolution. Models of cultural evolution (Richerson and Boyd, 2005) are crucially important for understanding religion. Human evolution is *co*evolution with cultural evolution mediated largely by processes of social learning, language, symbols, norms and religion.

The problem that every cooperating group faces is free riders-those who extract benefits of the group without actually contributing any resources to the group. In small groups we can keep track of free-riders via gossip, memory of cheats, reputations etc. However, as group size expands to modern-day societies that number in the billions, the ability for cognitive systems to track each potential cooperation partner via reputation falls apart. Group rituals may provide supplementary information via signaling mechanisms during the ritual as to who is cooperating and who is not (Whitehouse et al., 2014). At a cultural group selection level, groups that are formed on a basis of ritual demonstrations of devotion to the group are empirically observed to last longer, suggesting a direct selective benefit to forming social systems based in ritual performance (Sosis, 2006). But little or nothing is known about the brain bases of these signaling processing that occur during group rituals. Rituals must reinforce some mechanism that can inhibit free-riding without the need for individuals to track the free riders. One way to do this is to implant in each group member’s brain an internal-policemen or supernatural monitor who can observe and keep track of all your sins/free-riding. That internal implant equates to an authoritarian God image. As we have seen above the REM dreaming system could be co-opted to bias its output into generating and reinforcing such authoritarian God images. When combined with belief supernatural monitoring (especially if that monitoring is omniscient) of moral behavior, supernatural punishment beliefs that include daily/nightly reinforced images of an authoritarian God may inhibit some amount of free-riding and at a group level create a cluster of beliefs in Gods that are not only powerful but are also passionately interested in our “moral” behaviors (Norenzayan et al., 2016). An individual would need to monitor his or her relationship with the religious group in order to know if he or she was “sinning.” But again little or nothing is known about brain mechanisms that would mediate an individual’s interactions with the group.

**Figure 4:**
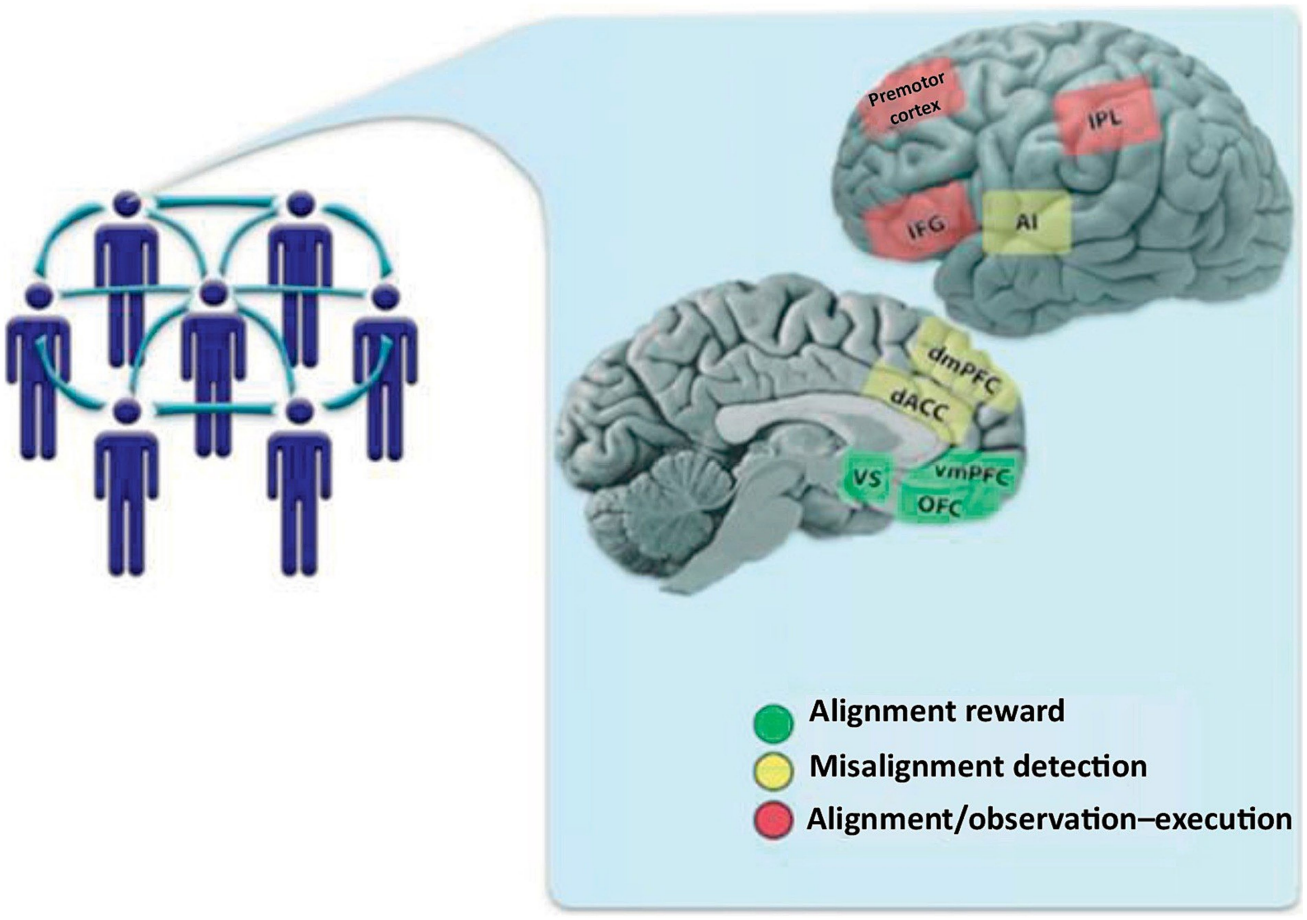
Brain mediation of individual alignment with group beliefs (used with permission from Shamay-Tsoory et al., 2019). The alignment system (red) comprises the inferior frontal gyrus (IFG), the inferior parietal lobule (IPL), and the premotor cortex. The alignment reward circuit (green) comprises the orbitofrontal cortex (OFC), the ventromedial PFC (vmPFC), and the ventral striatum (VS). When a gap between individual vs. group beliefs occurs it is detected/registered and then motivational and motor systems are engaged to realign and finally reward/salience systems reinforce that realignment.

Balch et al. (2023) suggested that brain mechanisms activated when an individual needs to-realign with group moral and religious norms are mediated via DMN and SN networks, particularly the anterior cingulate cortex (ACC) which is typically engaged in error detection and self-identity. Such an individual/group alignment brain mechanism was outlined by Shamay-Tsoory et al. (2019) and comprises the TPM canonical networks including the dorsomedial prefrontal cortex (dmPFC), the dorsal anterior cingulate cortex (dACC), and the anterior insula (AI). In Figure 3 the alignment system (red) comprises the inferior frontal gyrus (IFG), the inferior parietal lobule (IPL), and the premotor cortex. The alignment reward circuit (green) comprises the orbitofrontal cortex (OFC), the ventromedial PFC (vmPFC), and the ventral striatum (*VS*). When a gap between individual vs. group beliefs occurs it is detected/registered and then motivational and motor systems are engaged to realign and finally reward/salience systems reinforce that realignment (see Figure 4).

Cognitive neuroscience of religion projects can build upon the Shamay-Tsoory model of group alignment processes to include religion as a grouping variable in order to isolate brain mechanisms that place constraints on such individual vs. group interactions. Some recent studies have also linked brain oxytocin activity to group-level cooperative activities. For example, using allele trajectory modeling with both ancient and contemporary whole genomes, Joseph et al. (2024) showed that genes linked with increased oxytocinergic receptor or OXTR expression in the dopaminergic nucleus accumbens circuit or NAC and the dorsal anterior cingulate cortex or dACC (nodes in SN and DMN), as well as those linked with increased oxytocin (OT) secretion, underwent rapid positive selection in the Andes during a period of great societal and religious change over 2 thousand years ago. The timing and strength of the selection events suggest that the genetic effects of selection for cooperation, coordination, and social bonding may have played an outsized role compared with other possible drivers of selection like intergroup conflict. These selection events commenced around 2.5 and 1.25 thousand years ago, placing them in the region’s Upper Formative and Tiwanaku periods—a time of population growth, religious change (Janusek, 2006), urbanization, and relatively low rates of violence.

**Figure 5:**
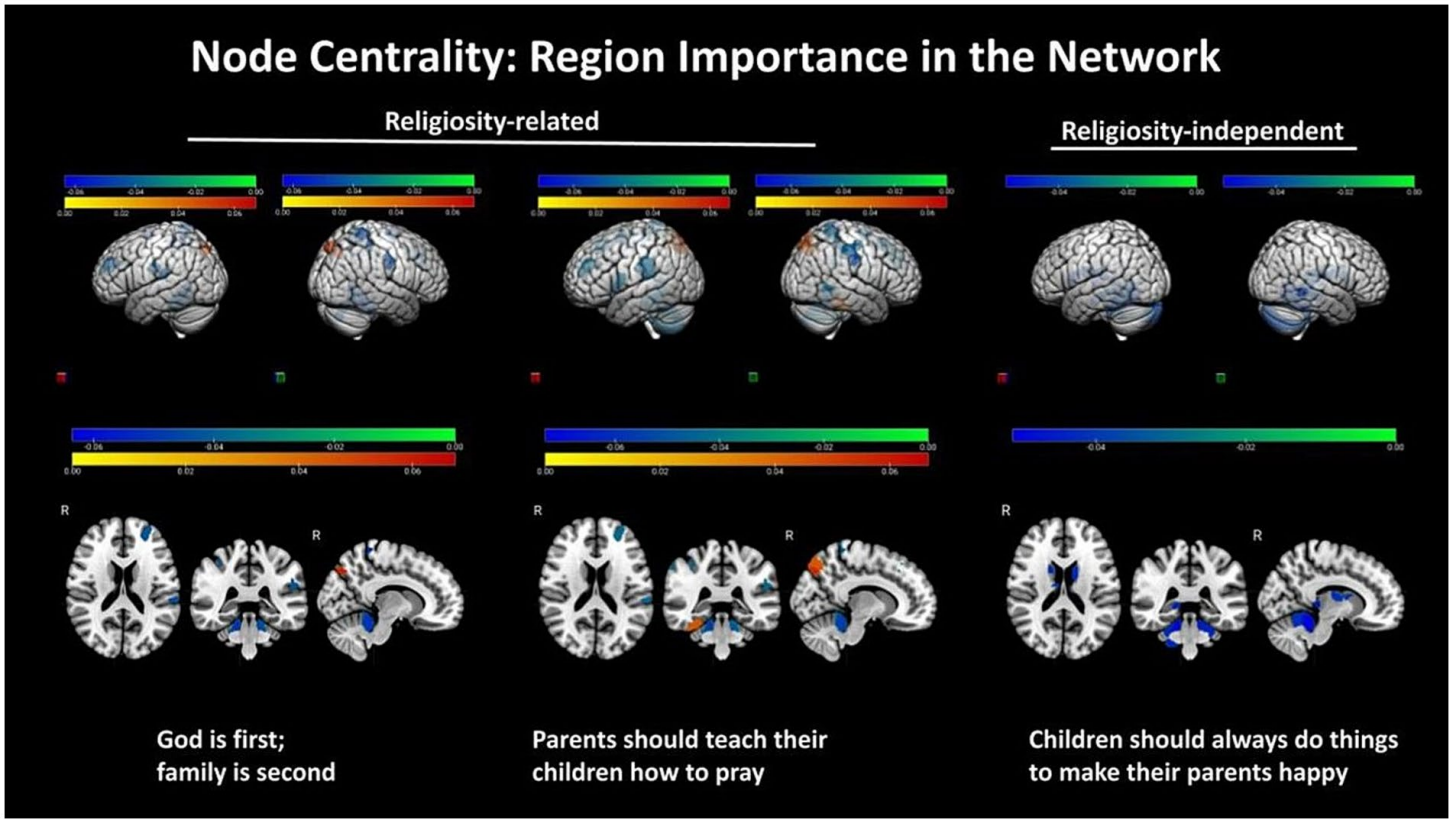
Node centrality: region importance in the network (used with permission from Brooks et al., 2022). Significant positive and negative associations were estimated, between strength of religious (God is first, family is second, and parents should teach their children how to pray), one religion-independent belief (children should always do things to make their parent happy), and node centrality (regional importance in a network). The colored bars represent the range of positive (yellow–red) and negative (green–blue) standardized regression coefficient values in statistical models that assessed these effects. Two and three-dimensional views of both hemispheres are shown.

We think another promising area for future research in the religion and brain landscape concerns developmental effects of religion on brain and vice versa. Typically developing neural circuits are highly heterogeneous. Brooks et al. (2022) studied such developmental effects in *N* = 5,566 children (median age = 120.0 months; 52.1% females; 71.2% with religious affiliation) from the Adolescent Brain Cognitive Development study. They examined relationships between parental religious and non-religious beliefs on the child’s TPM brain networks. Strength of both belief types was correlated with robustness of frontoparietal control, temporoparietal, and dorsal attention networks. Stronger religious beliefs were negatively associated with various properties of TPM-related salience, FPN and default-mode networks. While the findings were complex, they seem to conclusively demonstrate that religious beliefs of parents have measurable effects on their children’s brain functions and those effects are largely located within the TPM systems (see Figure 5).

Another example where religion and brain studies can benefit are in-depth clinical neuroscience studies of neuropsychiatric populations that evidence RSE changes. For example, there is some evidence that RSE measures change reliably in a small subgroup of patients with Parkinson’s Disease (PD). PD is a progressive neurodegenerative disorder characterized by both motor and the nonmotor deficits due, in part, to degeneration of dopaminergic neurons in the striatal and the mesolimbic-prefrontal dopaminergic systems. Among the non-motor deficits of PD, automatic access to religious representations may be slowed relative to controls particularly in a subgroup of PD patients-namely-left-onset patients (Butler et al., 2010). These patients also report fewer religious experiences and engage in fewer R/S practices than controls. These findings have recently been extended via examination of longitudinal relationships between R/S and PD onset (see Otaiku, 2023). Otaiku (2023) used multivariate logistic regression modeling on 2 longitudinal datasets to assess Parkinson’s Disease or PD-R/S associations across time. Analyses revealed that low religiosity at baseline was associated with a remarkable 10-fold increase in risk of developing PD many years later relative to individuals who reported higher religiosity at baseline. There was also evidence for a dose–response relation between religiosity level at baseline and later risk for PD. It is possible that years before onset of motor symptoms (and therefore diagnosis of PD) the NACc -meso-limbic dopaminergic and striatal-prefrontal dopaminergic systems begin to slowly degenerate thereby causing subtle personality changes including RSEs.

A final area where religion and brain studies could make a contribution is the rising probability that some AI (artificial intelligent) systems will begin to be treated as religious SAs that human beings defer to, reverence or submit to. What brain mechanisms permit or promote such behavior? The TPM cycling account might help to illuminate this kind of religious behavior directed at superintelligent machines if those machines are seen as generating a surplus of meaning-making opportunities. Recall that the TPM cycling account treats the canonical resting state networks as essentially a set of networks that assess significance, meaning and salience for self and the group. It would be surprising if these networks were not mediating interactions with these machines.

## Discussion

We find that at a broad network neuroscience level, religious/spiritual experiences (RSEs) appear to depend crucially upon interactions between the default mode network (DMN), the frontoparietal network (FPN), and the salience network (SN). We see this general result as broadly consistent with Menon, 2011 and Menon et al., 2023 “Triple Network or Tripartite Model” (TPM) of neuropsychiatric function/dysfunction. A TPM cycling model can account for the neural bases of an array of RSE phenomena including ecstatic seizures, neuroimaging of religious participants, psychedelically induced mystical states and perceptions of supernatural agents. To adequately account for SA perceptions, however, REM sleep and dreaming mechanisms likely also play a role. Future research should examine neurodevelopmental mechanisms of acquired SA perceptions as well as societal-level effects such as brain mediated religious beliefs of in-group cohesion and out-group hostility.

## Data Availability

The original contributions presented in the study are included in the article/supplementary material, further inquiries can be directed to the corresponding author.

## References

[ref1] Anand A. Barkay G. Dzemidzic M. Albrecht D. Karne H. Zheng Q.-H.. (2011). Striatal dopamine transporter availability in unmedicated bipolar disorder. Bipolar Disord. 13, 406–413. doi: 10.1111/j.1399-5618.2011.00936.x, PMID: 21843280

[ref2] Andersen M. (2019). Predictive coding in agency detection. Relig., Brain Behav. 9, 65–84. doi: 10.1080/2153599X.2017.1387170

[ref3] Ashok A. H. Marques T. R. Jauhar S. Nour M. M. Goodwin G. M. Young A. H.. (2017). The dopamine hypothesis of bipolar affective disorder: the state of the art and implications for treatment. Mol. Psychiatry 22, 666–679. doi: 10.1038/mp.2017.16, PMID: 28289283 PMC5401767

[ref4] Balch J. Grafman J. McNamara P. (2023). Finding consonance: an integrative neurocognitive model of human relationships with supernatural agents. Relig., Brain Behav. 14, 23–49. doi: 10.1080/2153599X.2022.2143398

[ref5] Banovac I. Sedmak D. Judaš M. Petanjek Z. (2021). Von Economo neurons – primate-specific or commonplace in the mammalian brain? Front. Neural Circ. 15:714611. doi: 10.3389/fncir.2021.714611, PMID: 34539353 PMC8440978

[ref6] Bear D. M. Fedio P. (1977). Quantitative analysis of interictal behavior in temporal lobe epilepsy. Arch. Neurol. 34, 454–467. doi: 10.1001/archneur.1977.00500200014003 889477

[ref7] Beaulieu J. M. Gainetdinov R. R. (2011). The physiology, signaling, and pharmacology of dopamine receptors. Pharmacol. Rev. 63, 182–217. doi: 10.1124/pr.110.002642 21303898

[ref8] Bellah R. N. (2011). Religion in human evolution: From the Paleolithic to the axial age Cambridge, MA: Harvard University Press.

[ref9] Block N. Miller B. (2020). “Religion and frontotemporal dementia” in Neurology and religion. eds. Coles A. Collicult J. (Cambridge, England: Cambridge University Press), 161–170.

[ref10] Brooks S. J. Tian L. Parks S. M. Stamoulis C. (2022). Parental religiosity is associated with changes in youth functional network organization and cognitive performance in early adolescence. Sci. Rep. 12:17305. doi: 10.1038/s41598-022-22299-6, PMID: 36243789 PMC9569366

[ref11] Brunello N. Tascedda F. (2003). Cellular mechanisms and second messengers: relevance to the psychopharmacology of bipolar disorders. Int. J. Neuropsychopharmacol. 6, 181–189. doi: 10.1017/S1461145703003419, PMID: 12890311

[ref12] Butler P. M. McNamara P. Durso R. (2010). Deficits in the automatic activation of religious concepts in patients with Parkinson’s disease. J. Int. Neuropsychol. Soc. 16, 252–261. doi: 10.1017/S1355617709991202, PMID: 19958570

[ref13] Carhart-Harris R. L. Erritzoe D. Williams T. Stone J. M. Reed L. J. Colasanti A.. (2012). Neural correlates of the psychedelic state as determined by fMRI studies with psilocybin. Proc. Natl. Acad. Sci. 109, 2138–2143. doi: 10.1073/pnas.1119598109, PMID: 22308440 PMC3277566

[ref14] Carhart-Harris R. L. Nutt D. (2017). Serotonin and brain function: a tale of two receptors. J. Psychopharmacol. 31, 1091–1120. doi: 10.1177/0269881117725915, PMID: 28858536 PMC5606297

[ref15] Chan D. Anderson V. Pijnenburg Y. Whitwell J. Barnes J. Scahill R.. (2009). The clinical profile of right temporal lobe atrophy. Brain 132, 1287–1298. doi: 10.1093/brain/awp037 19297506

[ref16] Chen Y. Zhang J. (2021). How energy supports our brain to yield consciousness: insights from neuroimaging based on the neuroenergetics hypothesis. Front. Syst. Neurosci. 15:648860. doi: 10.3389/fnsys.2021.648860, PMID: 34295226 PMC8291083

[ref17] Cheyne J. A. Rueffer S. D. Newby-Clark I. R. (1999). Hypnagogic and hypnopompic hallucinations during sleep paralysis: neurological and cultural construction of the night-mare. Conscious. Cogn. 8, 319–337. doi: 10.1006/ccog.1999.0404, PMID: 10487786

[ref18] Chow H. M. Horovitz S. G. Carr W. S. Picchioni D. Coddington N. Fukunaga M.. (2013). Rhythmic alternating patterns of brain activity distinguish rapid eye movement sleep from other states of consciousness. Proc. Natl. Acad. Sci. USA 110, 10300–10305. doi: 10.1073/pnas.1217691110, PMID: 23733938 PMC3690889

[ref19] Churchland P. S. Winkielman P. (2012). Modulating social behavior with oxytocin: how does it work? What does it mean? Horm. Behav. 61, 392–399. doi: 10.1016/j.yhbeh.2011.12.003, PMID: 22197271 PMC3312973

[ref20] Crespi B. J. (2016). Oxytocin, testosterone, and human social cognition. Biol. Rev. Camb. Philos. Soc. 91, 390–408. doi: 10.1111/brv.12175, PMID: 25631363

[ref21] Cristofori I. Cohen-Zimerman S. Bulbulia J. Gordon B. Krueger F. Grafman J. (2022). The neural underpinning of religious beliefs: evidence from brain lesions. Front. Behav. Neurosci. 16:977600. doi: 10.3389/fnbeh.2022.977600, PMID: 36275851 PMC9583670

[ref22] Dang-Vu T. T. Schabus M. Desseilles M. Sterpenich V. Bonjean M. Maquet P. (2010). Functional neuroimaging insights into the physiology of human sleep. Sleep 33, 1589–1603. doi: 10.1093/sleep/33.12.1589, PMID: 21120121 PMC2982729

[ref23] Davis A. K. Clifton J. M. Weaver E. G. Hurwitz E. S. Johnson M. W. Griffiths R. R. (2020). Survey of entity encounter experiences occasioned by inhaledN,N-dimethyltryptamine: phenomenology, interpretation, and enduring effects. J. Psychopharmacol. 34, 1008–1020. doi: 10.1177/0269881120916143, PMID: 32345112

[ref24] Dewhurst K. Beard A. W. (1970). Sudden religious conversions in temporal lobe epilepsy. Br. J. Psychiatry 117, 497–507. doi: 10.1192/bjp.117.540.497 5480697

[ref25] Dezutter J. Luyckx K. Schaap-Jonker H. Bussing A. Corveleyn J. Hutsebaut D. (2010). God image and happiness in chronic pain patients: the mediating role of disease interpretation. Pain Med. 11, 765–773. doi: 10.1111/j.1526-4637.2010.00827.x 20353410

[ref26] Dornisch S. J. (2024). Evolutionary perspectives on the association between religiosity and mental health: Organizing theoretical frameworks. Evol. Behav. Sci. doi: 10.1037/ebs0000364

[ref27] Duerler P. Vollenweider F. X. Preller K. H. (2022). A neurobiological perspective on social influence: serotonin and social adaptation. J. Neurochem. 162, 60–79. doi: 10.1111/jnc.15607, PMID: 35274296 PMC9322456

[ref28] Dunbar R. (2020). Religion, the social brain, and the mystical stance. Arch. Psychol. Relig. 42, 46–62. doi: 10.1177/0084672419900547

[ref29] Ferguson M. A. Schaper F. Cohen A. Siddiqi S. Merrill S. M. Nielsen J. A.. (2022). A neural circuit for spirituality and religiosity derived from patients with brain lesions. Biol. Psychiatry 91, 380–388. doi: 10.1016/j.biopsych.2021.06.016, PMID: 34454698 PMC8714871

[ref30] Fox K. C. R. Nijeboer S. Solomonova E. Domhoff G. W. Christoff K. (2013). Dreaming as mind wandering: evidence from functional neuroimaging and first-person content reports. Front. Hum. Neurosci. 7:412. doi: 10.3389/fnhum.2013.00412, PMID: 23908622 PMC3726865

[ref31] Francis L. J. (2007). “God images and empathy: a study among secondary school pupils in England” in What do we imagine god to be? The function of ‘god images’ in our lives. ed. Heggy P. (Lewiston, NY: Edwin Mellen Press), 67–88.

[ref32] Froese P. Bader C. (2008). Unraveling religious worldviews: the relationship between images of god and political ideology in a cross-cultural analysis. Sociol. Q. 49, 689–718. doi: 10.1111/j.1533-8525.2008.00132.x

[ref33] Gall T. L. Guirguis-Younger M. (2013). “Religious and spiritual coping: current theory and research” in APA handbook of psychology, religion, and spirituality. eds. Pargament K. I. Exline J. J. Jones J. W., vol. 1 (Washington, DC: American Psychological Association), 349–364.

[ref34] Gattuso J. J. Perkins D. Ruffell S. Lawrence A. J. Hoyer D. Jacobson L. H.. (2023). Default mode network modulation by psychedelics: a systematic review. Int. J. Neuropsychopharmacol. 26, 155–188. doi: 10.1093/ijnp/pyac074, PMID: 36272145 PMC10032309

[ref35] Geschwind N. (1979). Behavioral changes in temporal lobe epilepsy. Psychol. Med. 9, 217–219. doi: 10.1017/S0033291700030713, PMID: 472070

[ref36] Grafman J. Cristofori I. Zhong W. Bulbulia J. (2020). The neural basis of religious cognition. Curr. Dir. Psychol. Sci. 29, 126–133. doi: 10.1177/0963721419898183

[ref37] Griffiths R. R. Hurwitz E. S. Davis A. K. Johnson M. W. Jesse R. (2019). Survey of subjective “god encounter experiences”: comparisons among naturally occurring experiences and those occasioned by the classic psychedelics psilocybin, LSD, ayahuasca, or DMT. PLoS One 14:e0214377. doi: 10.1371/journal.pone.0214377, PMID: 31013281 PMC6478303

[ref38] Gschwind M. Picard F. (2016). Ecstatic epileptic seizures: a glimpse into the multiple roles of the insula. Front. Behav. Neurosci. 10:21. doi: 10.3389/fnbeh.2016.00021, PMID: 26924970 PMC4756129

[ref39] Halman A. Kong G. Sarris J. Perkins D. (2024). Drug–drug interactions involving classic psychedelics: a systematic review. J. Psychopharmacol. 38, 3–18. doi: 10.1177/02698811231211219, PMID: 37982394 PMC10851641

[ref40] He J. Bore M. C. Jiang H. Gan X. Wang J. Li J.. (2024). Neural basis of pain empathy dysregulations in mental disorders–a pre-registered neuroimaging meta-analysis. Biol. Psychiatry Cogn Neurosci Neuroimaging. doi: 10.1016/j.bpsc.2024.08.019, PMID: 39260566

[ref41] Hefnawy M. T. Amer B. E. Amer S. A. Moghib K. Khlidj Y. Elfakharany B.. (2024). Prevalence and clinical characteristics of sleeping paralysis: a systematic review and meta-analysis. Cureus 16:e53212. doi: 10.7759/cureus.53212, PMID: 38425633 PMC10902800

[ref42] Hishikawa Y. (1976). “Sleep paralysis” in Advances in sleep research, Ed., Elliot D. Weitzman vol. 3 (New York, NY: Academic Press), 97–124.

[ref43] Hobson J. A. Pace-Schott E. F. Stickgold R. (2000). Dreaming and the brain: toward a cognitive neuroscience of conscious states. Behav. Brain Sci. 23, 793–842. doi: 10.1017/S0140525X00003976, PMID: 11515143

[ref44] Holbrook C. Iacoboni M. Gordon C. Proksch S. Balasubramaniam R. (2020). Posterior medial frontal cortex and threat-enhanced religious belief: a replication and extension. Soc. Cogn. Affect. Neurosci. 15, 1361–1367. doi: 10.1093/scan/nsaa153, PMID: 33180108 PMC7759203

[ref45] Holliday M. A. (1986). “Body composition and energy needs during growth” in Postnatal growth neurobiology. eds. Falkner F. Tanner J. M. (Boston, MA: Springer), 1–20.

[ref46] Horowitz-Kraus T. Farah R. Hajinazarian A. Eaton K. Rajagopal A. Schmithorst V. J.. (2017). Maturation of brain regions related to the default mode network during adolescence facilitates narrative comprehension. J. Child Adoles. Behav. 5:328. doi: 10.4172/2375-4494.1000328, PMID: 32524005 PMC7286598

[ref47] Hughes J. R. Melyn M. (2005). EEG and seizures in autistic children and adolescents: further findings with therapeutic implications. Clin. EEG Neurosci. 36, 15–20. doi: 10.1177/155005940503600105, PMID: 15683193

[ref48] Hutchison I. C. Rathore S. (2015). The role of REM sleep theta activity in emotional memory. Front. Psychol. 6:1439. doi: 10.3389/fpsyg.2015.01439, PMID: 26483709 PMC4589642

[ref49] Janusek J. W. (2006). The changing ‘nature’ of Tiwanaku religion and the rise of an Andean state. World Archaeol. 38, 469–492. doi: 10.1080/00438240600813541

[ref50] Johnson D. D. P. (2016). God is watching you: How the fear of god makes us human: Oxford, England: Oxford University Press.

[ref51] Joseph S. K. Wagman E. Diab N. Ryu N. Lee M. Haas R.. (2024). Paleogenomic insights into cooperation in the ancient Andes from positive selection on oxytocin pathway genes. Genes Brain Behav. 23:e12877. doi: 10.1111/gbb.12877

[ref52] Kapogiannis D. Barbey A. K. Su M. Krueger F. Grafman J. (2009). Neuroanatomical variability of religiosity. PLoS One 4:7180. doi: 10.1371/journal.pone.0007180, PMID: 19784372 PMC2746321

[ref53] Kapogiannis D. Deshpande G. Krueger F. Thornburg M. P. Grafman J. H. (2014). Brain networks shaping religious belief. Brain Connect. 4, 70–79. doi: 10.1089/brain.2013.0172, PMID: 24279687 PMC3929007

[ref54] Kelly J. M. Kramer S. R. Shariff A. F. (2024). Religiosity predicts prosociality, especially when measured by self-report: a meta-analysis of almost 60 years of research. Psychol. Bull. 150, 284–318. doi: 10.1037/bul0000413, PMID: 38407059

[ref55] Kieckhaefer C. Schilbach L. Bzdok D. (2023). Social belonging: brain structure and function is linked to membership in sports teams, religious groups, and social clubs. Cereb. Cortex 33, 4405–4420. doi: 10.1093/cercor/bhac351, PMID: 36161309 PMC10110433

[ref56] Koenig H. G. VanderWeele T. J. Peteet J. R. (2024). Handbook of religion and health Oxford, England: Oxford University Press.

[ref57] Krueger F. Grafman J. (Eds.) (2012). The neural basis of human belief systems: New York, NY: Psychology Press.

[ref58] Landolt H. Wehrle R. (2009). Antagonism of serotonergic 5-HT2A/2C receptors: mutual improvement of sleep, cognition, and mood? Eur. J. Neurosci. 29, 1795–1809. doi: 10.1111/j.1460-9568.2009.06718.x, PMID: 19473234

[ref59] Lebedev A. V. Lövdén M. Rosenthal G. Feilding A. Nutt D. J. Carhart-Harris R. L. (2015). Finding the self by losing the self: neural correlates of ego-dissolution under psilocybin. Hum. Brain Mapp. 36, 3137–3153. doi: 10.1002/hbm.22833, PMID: 26010878 PMC6869189

[ref60] Llewellyn S. (2016). Dream to predict? Dreaming as predictive coding. Front. Psychol. 7:1961. doi: 10.3389/fpsyg.2016.01961, PMID: 26779078 PMC4700581

[ref61] Luppi P.-H. Billwiller F. Fort P. (2017). Selective activation of a few limbic structures during paradoxical (REM) sleep by the claustrum and the supramammillary nucleus: evidence and function. Curr. Opin. Neurobiol. 44, 59–64. doi: 10.1016/j.conb.2017.03.002, PMID: 28347885

[ref62] Maquet P. Peters J.-M. Aerts J. Delfiore G. Degueldre C. Luxen A.. (1996). Functional neuroanatomy of human rapid-eye-movement sleep and dreaming. Nature 383, 163–166. doi: 10.1038/383163a0, PMID: 8774879

[ref63] McNamara P. (2022). The cognitive neuroscience of religious experience: decentering and the self Cambridge, England: Cambridge University Press.

[ref64] Mencken F. Carson C. D. Bader C. Embry E. (2009). In god we trust: images of god and trust in the United States among the highly religious. Sociol. Perspect. 52, 23–38. doi: 10.1525/sop.2009.52.1.23

[ref65] Menon V. (2011). Large-scale brain networks and psychopathology: a unifying triple network model. Trends Cogn. Sci. 15, 483–506. doi: 10.1016/j.tics.2011.08.003, PMID: 21908230

[ref66] Menon V. Palaniyappan L. Supekar K. (2023). Integrative brain network and salience models of psychopathology and cognitive dysfunction in schizophrenia. Biol. Psychiatry 94, 108–120. doi: 10.1016/j.biopsych.2022.09.029, PMID: 36702660

[ref67] Mulders P. C. van Eijndhoven P. F. Schene A. H. Beckmann C. F. Tendolkar I. (2015). Resting-state functional connectivity in major depressive disorder: a review. Neurosci. Biobehav. Rev. 56, 330–344. doi: 10.1016/j.neubiorev.2015.07.014 26234819

[ref68] Nencha U. Spinelli L. Vulliemoz S. Seeck M. Picard F. (2022). Insular stimulation produces mental clarity and bliss. Ann. Neurol. 91, 289–292. doi: 10.1002/ana.26282, PMID: 34877703 PMC9300149

[ref69] Nguyen T. T. Bellehumeur C. Malette J. (2015). God images and resilience: a study of Vietnamese immigrants. J. Psychol. Theol. 43, 271–282. doi: 10.1177/009164711504300405

[ref70] Nofzinger E. A. Mintun M. A. Wiseman M. B. Kupfer D. J. Moore R. Y. (1997). Forebrain activation in REM sleep: an FDG PET study. Brain Res. 770, 192–201. doi: 10.1016/S0006-8993(97)00807-X, PMID: 9372219

[ref71] Norenzayan A. (2013). Big gods: How religion transformed cooperation and conflict Princeton, NJ: Princeton University Press.

[ref72] Norenzayan A. Shariff A. F. Gervais W. M. Willard A. K. McNamara R. A. Slingerland E.. (2016). The cultural evolution of prosocial religions. Behav. Brain Sci. 39:e1. doi: 10.1017/S0140525X14001356, PMID: 26785995

[ref73] Otaiku A. I. (2023). Religiosity and risk of Parkinson’s disease in England and the USA. J. Relig. Health 62, 4192–4208. doi: 10.1007/s10943-022-01603-8, PMID: 35763200 PMC10682218

[ref74] Ouwehand E. (2024). Religious experiences in the context of bipolar disorder: serious pathology and/or genuine spirituality? A narrative review against the background of the literature about bipolar disorder and religion. Religions 15:274. doi: 10.3390/rel15030274

[ref75] Pace-Schott E. F. Hobson J. A. (2002). The neurobiology of sleep: genetics, cellular physiology and subcortical networks. Nat. Rev. Neurosci. 3, 591–605. doi: 10.1038/nrn895, PMID: 12154361

[ref76] Pace-Schott E. F. Picchioni D. (2017). “The neurobiology of dreaming” in Principles and practice of sleep medicine. eds. Kryger M. H. Roth T. Dement W. C.. 6th ed (Philadelphia: Elsevier), 529–538.

[ref77] Park C. L. (2005). “Religion and meaning” in Handbook of the psychology of religion and spirituality. eds. Park C. L. Palmer A. L. K. (New York, NY: Guilford Press), 357–379.

[ref78] Park H. D. Blanke O. (2019). Coupling inner and outer body for self-consciousness. Trends Cogn. Sci. 23, 377–388. doi: 10.1016/j.tics.2019.02.002 30826212

[ref79] Park S. H. Weber F. (2020). Neural and homeostatic regulation of REM sleep. Front. Psychol. 11:1662. doi: 10.3389/fpsyg.2020.01662, PMID: 32793050 PMC7385183

[ref80] Pew Research Center. (2022). Key findings from the global religious futures project. Pew Research Center. Available at: https://www.pewresearch.org/religion/2022/12/21/key-findings-from-the-global-religious-futures-project/

[ref81] Preller K. H. Vollenweider F. X. (2018). “Phenomenology, structure, and dynamic of psychedelic states” in Current topics in behavioral neurosciences. eds. Halberstadt A. L. Vollenweider F. X. Nichols D. E. (Cham, Switzerland: Springer), 221–256. 10.1007/7854_2016_459 28025814

[ref82] Pyysiainen I. (2009). Supernatural agents: Why we believe in souls, gods, and Buddhas. Oxford: Oxford University Press.

[ref83] Rabitti E. Cavuto S. Díaz Crescitelli M. E. Bassi M. C. Ghirotto L. (2024). Hospice patients’ end-of-life dreams and visions: a systematic review of qualitative studies. Am. J. Hosp. Palliat. Med. 41, 99–112. doi: 10.1177/10499091231163571, PMID: 36947427 PMC10710003

[ref84] Richerson P. J. Boyd R. (2005). Not by genes alone: how culture transformed human evolution Chicago, IL: University of Chicago Press.

[ref85] Ronchi R. Park H. D. Blanke O. (2018). “Bodily self-consciousness and its disorders” in Handbook of clinical neurology, vol. 151 (Amsterdam, Netherlands: Elsevier), 313–330. 29519466 10.1016/B978-0-444-63622-5.00015-2

[ref86] Rosmarin D. H. Kaufman C. C. Ford S. F. Keshava P. Drury M. Minns S.. (2022). The neuroscience of spirituality, religion, and mental health: a systematic review and synthesis. J. Psychiatr. Res. 156, 100–113. doi: 10.1016/j.jpsychires.2022.10.003, PMID: 36244198

[ref87] Saleh T. Logothetis N. Evrard H. (2017). Insular projections to brainstem homeostatic centers in the macaque monkey. Front. Mol. Neurosci. 10:11. doi: 10.3389/fnmol.2017.00011, PMID: 28179877 PMC5263138

[ref88] Schaap-Jonker H. Eurelings-Bontekoe E. Verhagen P. J. Zock H. (2002). Image of god and personality pathology: an exploratory study among psychiatric patients. Mental Health Relig. Cult. 5, 55–71. doi: 10.1080/13674670110112712

[ref89] Schjoedt U. (2009). The religious brain: a general introduction to the experimental neuroscience of religion. Method Theory Study Relig. 21, 310–339. doi: 10.1163/157006809X460347

[ref90] Seeley W. W. Menon V. Schatzberg A. F. Keller J. Glover G. H. Kenna H.. (2007). Dissociable intrinsic connectivity networks for salience processing and executive control. J. Neurosci. 27, 2349–2356. doi: 10.1523/JNEUROSCI.5587-06.2007 17329432 PMC2680293

[ref91] Seitz R. J. Paloutzian R. F. Angel H. F. (2018). From believing to belief: a general theoretical model. J. Cogn. Neurosci. 30, 1254–1264. doi: 10.1162/jocn_a_01292 29877765

[ref92] Sfera A. Hassan I. Bota P. (2024). Frontotemporal dementia behavioral variant as an acquired psychopathy. Modern Neurosci. 1, 5–8. doi: 10.55828/mnn-11-02

[ref93] Sha Z. Wager T. D. Mechelli A. He Y. (2019). Common dysfunction of large-scale neurocognitive networks across psychiatric disorders. Biol. Psychiatry 85, 379–388. doi: 10.1016/j.biopsych.2018.11.011, PMID: 30612699

[ref94] Shamay-Tsoory S. G. Abu-Akel A. (2016). The social salience hypothesis of oxytocin. Biol. Psychiatry 79, 194–202. doi: 10.1016/j.biopsych.2015.07.020 26321019

[ref95] Shamay-Tsoory S. G. Saporta N. Marton-Alper I. Z. Gvirts H. Z. (2019). Herding brains: a core neural mechanism for social alignment. Trends Cogn. Sci. 23, 174–186. doi: 10.1016/j.tics.2019.01.002, PMID: 30679099

[ref96] Shariff A. F. Norenzayan A. (2011). Mean gods make good people: different views of god predict cheating behavior. Int. J. Psychol. Relig. 21, 85–96. doi: 10.1080/10508619.2011.556990

[ref97] Shokri-Kojori E. Tomasi D. Alipanahi B. Wiers C. E. Wang G. J. Volkow N. D. (2019). Correspondence between cerebral glucose metabolism and BOLD reveals relative power and cost in human brain. Nature. Communications 10:690. doi: 10.1038/s41467-019-08546-x, PMID: 30741935 PMC6370887

[ref98] Shulman R. G. Hyder F. Rothman D. L. (2014). Insights from neuroenergetics into the interpretation of functional neuroimaging: an alternative empirical model for studying the brain’s support of behavior. J. Cereb. Blood Flow Metab. 34, 1721–1735. doi: 10.1038/jcbfm.2014.145, PMID: 25160670 PMC4269754

[ref99] Silton N. R. Flannelly K. J. Galek K. Ellison C. G. (2014). Beliefs about god and mental health among American adults. J. Relig. Health 53, 1285–1296. doi: 10.1007/s10943-013-9712-3, PMID: 23572240

[ref100] Sosis R. (2006). “Religious behaviors, badges, and bans: signaling theory and the evolution of religion” in Where god and science meet: How brain and evolutionary sciences alter our understanding of religion. ed. McNamara P., vol. I (Westport, CT: Praeger Press), 61–86.

[ref101] Sridharan D. Levitin D. J. Menon V. (2008). A critical role for the right fronto-insular cortex in switching between central-executive and default-mode networks. Proc. Nat. Acad. Sci. U.S.A. 105, 12569–12574. doi: 10.1073/pnas.0800005105, PMID: 18723676 PMC2527952

[ref102] Stockly K. (2020). Appendix B: how is god “like a drug”? Exploring the evolution of social affects and oxytocin in High on god: How megachurches won the heart of America. eds. Wellman J. Corcoran K. Stockly K. (New York, NY: Oxford University Press), 251–284. Available at: https://oxford-universitypressscholarship-com.ezproxy.bu.edu/view/10.1093/oso/9780199827718.001.0001/oso-9780199827718

[ref103] Stoliker D. Egan G. F. Friston K. J. Razi A. (2022). Neural mechanisms and psychology of psychedelic ego dissolution. Pharmacol. Rev. 74, 876–917. doi: 10.1124/pharmrev.121.000508, PMID: 36786290

[ref104] Tagliazucchi E. Roseman L. Kaelen M. Orban C. Muthukumaraswamy S. D. Murphy K.. (2016a). Increased global functional connectivity correlates with LSD-induced ego dissolution. Curr. Biol. 26, 1043–1050. doi: 10.1016/j.cub.2016.02.010, PMID: 27085214

[ref105] Tagliazucchi E. Roseman L. Kaelen M. Orban C. Muthukumaraswamy S. Murphy K.. (2016b). Increased global functional connectivity correlates with LSD-induced ego dissolution. Front. Neurosci. 16:827400. doi: 10.3389/fnins.2022.827400, PMID: 27085214

[ref106] Uddin L. Q. Menon V. (2009). The anterior insula in autism: under-connected and under-examined. Neurosci. Biobehav. Rev. 33, 1198–1203. doi: 10.1016/j.neubiorev.2009.06.002, PMID: 19538989 PMC2743776

[ref107] Van Elk M. Aleman A. (2017). Brain mechanisms in religion and spirituality: an integrative predictive processing framework. Neurosci. Biobehav. Rev. 73, 359–378. doi: 10.1016/j.neubiorev.2016.12.031, PMID: 28041787

[ref108] van Elk M. Yaden D. B. (2022). Pharmacological, neural, and psychological mechanisms underlying psychedelics: a critical review. Neurosci. Biobehav. Rev. 140:104793. doi: 10.1016/j.neubiorev.2022.104793, PMID: 35878791

[ref109] Vehar N. Potheegadoo J. Blanke O. (2022). Linking agent detection of invisible presences to the self: relevance for religious and spiritual experiences. Front. Behav. Neurosci. 16:952736. doi: 10.3389/fnbeh.2022.952736, PMID: 35836488 PMC9274283

[ref110] Veronelli L. Makaretz S. J. Quimby M. Dickerson B. C. Collins J. A. (2017). Geschwind syndrome in frontotemporal lobar degeneration: neuroanatomical and neuropsychological features over 9 years. Cortex 94, 27–38. doi: 10.1016/j.cortex.2017.06.003, PMID: 28711815 PMC5565695

[ref111] Vollenweider F. X. Preller K. H. (2020). Psychedelic drugs: neurobiology and potential for treatment of psychiatric disorders. Nat. Rev. Neurosci. 21, 611–624. doi: 10.1038/s41583-020-0367-2 32929261

[ref112] Walker M. P. Stickgold R. (2006). Sleep, memory, and plasticity. Annu. Rev. Psychol. 57, 139–166. doi: 10.1146/annurev.psych.56.091103.070307 16318592

[ref113] Waxman S. G. Geschwind N. (1975). The interictal behavior syndrome of temporal lobe epilepsy. Arch. Gen. Psychiatry 32, 1580–1586. doi: 10.1001/archpsyc.1975.01760300118011 1200777

[ref115] Whitehouse H. Lanman J. A. Downey G. Fredman L. A. Swann W. B. Jr. Lende D. H.. (2014). The ties that bind us: ritual, fusion, and identification. Curr. Anthropol. 55, 675–695. doi: 10.1086/678232

[ref116] Wilson D. S. Wilson E. O. (2007). Rethinking the theoretical foundation of sociobiology. Q. Rev. Biol. 82, 327–348. doi: 10.1086/522809, PMID: 18217526

[ref117] Wright S. T. Kerr C. W. Doroszczuk N. M. Kuszczak S. M. Hang P. C. Luczkiewicz D. L. (2013). The impact of dreams of the deceased on bereavement: a survey of hospice caregivers. Am. J. Hosp. Palliat. Med. 31, 132–138. doi: 10.1177/1049909113479201, PMID: 23449603

[ref118] Zhang W. Du J. L. Fang X. Y. Ni L. Y. Zhu Y. Y. Yan W.. (2023). Shared and distinct structural brain alterations and cognitive features in drug-naïve schizophrenia and bipolar disorder. Asian J. Psychiatr. 82:103513. doi: 10.1016/j.ajp.2023.103513, PMID: 36827938

[ref119] Zhao X. Ozols A. B. Meyers K. T. Campbell J. McBride A. Marballi K. K.. (2022). Acute sleep deprivation upregulates serotonin 2A receptors in the frontal cortex of mice via the immediate early gene Egr3. Mol. Psychiatry 27, 1599–1610. doi: 10.1038/s41380-021-01390-w, PMID: 35001075 PMC9210263

